# Alpine permafrost could account for a quarter of thawed carbon based on Plio-Pleistocene paleoclimate analogue

**DOI:** 10.1038/s41467-022-29011-2

**Published:** 2022-03-14

**Authors:** Feng Cheng, Carmala Garzione, Xiangzhong Li, Ulrich Salzmann, Florian Schwarz, Alan M. Haywood, Julia Tindall, Junsheng Nie, Lin Li, Lin Wang, Benjamin W. Abbott, Ben Elliott, Weiguo Liu, Deepshikha Upadhyay, Alexandrea Arnold, Aradhna Tripati

**Affiliations:** 1 Key Laboratory of Orogenic Belts and Crustal Evolution, Ministry of Education, School of Earth and Space Sciences, Peking University, Beijing, 100871 China; 2 Department of Earth and Environmental Sciences, University of Rochester, Rochester, NY 14627 USA; 3 Department of Environmental Sciences, Rochester Institute of Technology, Rochester, NY 14623 USA; 4 College of Science, University of Arizona, Tucson, AZ 85721 USA; 5 Yunnan Key Laboratory of Earth System Science, Yunnan University, Kunming, 650500 China; 6 State Key Laboratory of Loess and Quaternary Geology, Institute of Earth Environment, Chinese Academy of Science, Xi’an, 710061 China; 7 Department of Geography and Environmental Sciences, Northumbria University, Newcastle upon Tyne, NE1 8ST UK; 8 School of Earth and Environment, University of Leeds, Woodhouse Lane, Leeds, LS2 9JT UK; 9 Key Laboratory of Western China’s Environmental Systems (Ministry of Education), College of Earth and Environmental Sciences, Lanzhou University, Lanzhou, 730000 China; 10 Department of Civil and Environmental Engineering, The Hong Kong University of Science and Technology, Hong Kong SAR, China; 11 Department of Plant and Wildlife Sciences, Brigham Young University, Provo, UT USA; 12 Department of Earth, Planetary, and Space Sciences, Department of Atmospheric and Oceanic Sciences, Institute of the Environment and Sustainability, Center for Diverse Leadership in Science, University of California, Los Angeles, CA 90095 USA

**Keywords:** Palaeoclimate, Stratigraphy, Carbon cycle, Cryospheric science

## Abstract

Estimates of the permafrost-climate feedback vary in magnitude and sign, partly because permafrost carbon stability in warmer-than-present conditions is not well constrained. Here we use a Plio-Pleistocene lacustrine reconstruction of mean annual air temperature (MAAT) from the Tibetan Plateau, the largest alpine permafrost region on the Earth, to constrain past and future changes in permafrost carbon storage. Clumped isotope-temperatures (Δ_47_-T) indicate warmer MAAT (~1.2 °C) prior to 2.7 Ma, and support a permafrost-free environment on the northern Tibetan Plateau in a warmer-than-present climate. Δ_47_-T indicate ~8.1 °C cooling from 2.7 Ma, coincident with Northern Hemisphere glacial intensification. Combined with climate models and global permafrost distribution, these results indicate, under conditions similar to mid-Pliocene Warm period (3.3–3.0 Ma), ~60% of alpine permafrost containing ~85 petagrams of carbon may be vulnerable to thawing compared to ~20% of circumarctic permafrost. This estimate highlights ~25% of permafrost carbon and the permafrost-climate feedback could originate in alpine areas.

## Abstract (web summary)

The stability of permafrost carbon is poorly understood. Here the authors use Plio-Pleistocene clumped isotope reconstructions from the Tibetan Plateau and climate simulation to determine that ~85 petagrams of alpine carbon is vulnerable to thawing.

## Introduction

**Fig. 1:**
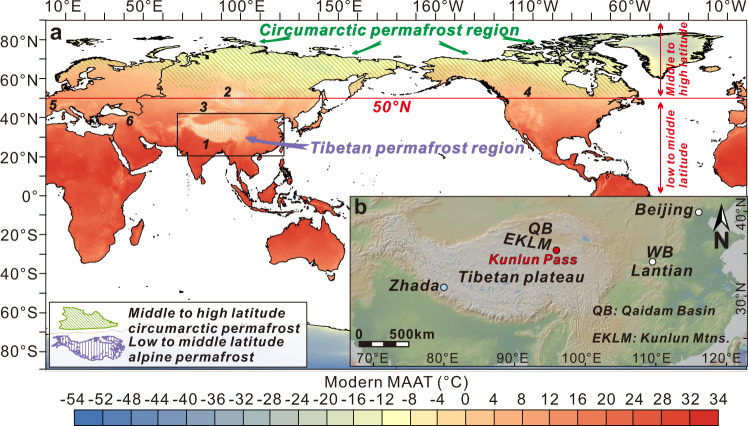
Global map showing modern mean annual air temperature, the distribution of permafrost, and inset with the location of the study area. **a** Modern mean annual air temperature map^70^ showing circumarctic and alpine permafrost^49^. **b** Topographic map of the Tibetan Plateau and surrounding regions showing the Kunlun Pass section on the northern Tibetan Plateau. QB: Qaidam basin, EKLM: Eastern Kunlun Mountains,WB: Weihe Basin. Note that regions north of 50°N are defined as middle to high latitude circumarctic regions. Region marked with 1–6 refers to the Tibetan Plateau-Pamir, Altai Mountains-Mongolia-Yablonoi-Sayan, Tian Shan, Rocky Mountains, Alps, and Caucasus, respectively. The image of landform made with GeoMapApp 3.6.10 (www.geomapapp.org)/CC BY.

Permafrost, or permanently frozen ground, underlies less than 20% of the Earth’s land area^1^. However, soils in the permafrost zone store ~1500 Pg (petagrams, 10^15^ grams) of organic carbon, representing ~50% of global soil organic carbon (SOC) and nearly twice as much carbon as contained by the Earth’s atmosphere^2–4^. While most modern permafrost is located in circumarctic regions, including subsea deposits on the continental shelves, permafrost extends through alpine areas at lower latitudes^1^ (Fig. 1a). Most of this alpine permafrost occurs on the Tibetan Plateau, which is sometimes referred to as Earth’s “Third Pole”, containing a globally significant stock of ~160 Pg of SOC^3,5^. Monitoring and modelling studies reveal that across the globe, permafrost is thawing rapidly^6,7^, giving rise to a potential permafrost-climate feedback. Once organic matter in permafrost is thawed, it can be decomposed by microorganisms, producing the greenhouse gases carbon dioxide (CO_2_), methane (CH_4_), and nitrous oxide (N_2_O)^8,9^. However, fundamental uncertainties persist about the response of permafrost carbon to climate change^10^, and modelling studies currently disagreeing on the magnitude and sign, of the permafrost-climate feedback^11,12^. In particular, determining regions (circumarctic versus alpine regions) where permafrost carbon is more sensitive to global warming is urgently needed.

The distribution of permafrost during past climatic warm periods could shed light on the near-future response of this system to anthropogenic forcing. Geological and modelling studies indicate that the Earth was warmer than present during the mid-Pliocene Warm Period (mPWP; 3.3–3.0 Ma), with atmospheric CO_2_ concentrations near 400 ppmv (parts per million by volume)^13,14^. The mPWP is often used as a geological analogue for the near future, to study climate change impacts on sea level and extent of glaciers and ice sheets. Considering that global climate will soon reach mPWP-like conditions if current anthropogenic warming continues^13^, paleoclimate reconstruction for the mPWP and Plio-Pleistocene in regions that today contain permafrost can provide insights into the stability of circumarctic and alpine permafrost^14^. Although over the last few decades, our knowledge of the mPWP and Plio-Pleistocene paleoclimate across the globe has improved significantly^15–17^, little is known about the climate history of regions that today contain permafrost, including both circumarctic and alpine permafrost zones. The lack of comprehensive paleoenvironmental studies in modern permafrost-containing regions also represents an important knowledge gap about the response of high elevation and high latitude regions to climate change.

Multiple geochemical proxies now allow detailed reconstruction of paleoclimate from marine and lacustrine sediments. Carbonate stable isotope (δ^18^O_c_ and δ^13^C_c_), carbonate content (CaCO_3_), total organic carbon (TOC), total nitrogen (TN), carbon to nitrogen ratios (C/N), carbon isotope ratios in organic matter (δ^13^C_org_), and grain size have been widely used for paleoenvironmental reconstruction as these proxies provide qualitative constraints on temperature, evaporation-precipitation balance, primary productivity, erosion and other climate conditions that might indirectly relate to temperature^18–20^. One particularly promising temperature proxy is the carbonate-clumped isotope (Δ_47_), thermometer, which can provide robust paleotemperature constraints independent of the oxygen isotopic composition of water (δ^18^O_w_)^21^. Given that the persistence of permafrost depends on temperature-ecosystem interactions^22,23^, reliable reconstructions of mean annual air temperatures (MAAT) from Plio-Pleistocene sediments would not only enable us to assess the presence of permafrost during the mPWP warmth, but also projects the modern permafrost regions that might be particularly vulnerable to future global warming. Comparing geological records from sparsely exposed outcrops or drilling core sites on the Tibetan Plateau with climate models simulations allows for paleoclimate reconstruction in a global context and also improves our understanding of the importance of mountain permafrost in future climate change scenarios^13^.

Here we estimate the potential area of global permafrost thaw, and associated organic carbon in a warmer-than-present climate, by integrating paleoclimate reconstructions from geological records with climate models to explore the implications of past changes in temperature on permafrost distributions. We used the Pliocene Model Intercomparison Project 2 (PlioMIP 2)^24^ model ensemble, which stands out for its ability to provide first-order global climatic conditions for the mPWP, to further estimate the stability of modern permafrost in a global context during the warmer climate of the mPWP. Specifically, we first reconstruct paleotemperature and other paleoenvironmental conditions in an alpine permafrost region on the northern Tibetan Plateau for the Pliocene to Pleistocene (from 4.3 to 0.8 Ma) from lacustrine records near Kunlun Pass (KP) (Fig. 1b). For this work, a total of 344 samples were collected and analysed for CaCO_3_, grain size, TN, TOC, C/N ratio, and δ^13^C_org._ A subset of 275 samples was also analysed for carbonate δ^18^O_c_ and δ^13^C_c_ analyses. 57 samples were analysed for Δ_47_ composition, which was used to estimate local MAAT, in conjunction with the other proxies. Our results reveal that a stepped cooling event occurred during the Plio-Pleistocene transition with warmer temperatures (MAAT > 0 °C) during the Pliocene in the northern Tibetan Plateau, indicating a potentially permafrost-free environment on the margins of the Tibetan Plateau. Using this inference as a guide, we then derived a mPWP global MAAT and mapped out the 0 °C isotherm using results from the Pliocene Model Intercomparison Project 2 (PlioMIP 2)^24^, which we also compared with the observed paleotemperature records from the KP site, to assess the stability of permafrost across the globe in a warmer-than-present world. By combining these estimates with the modern and pre-industrial global MAAT record, the modern global distribution of permafrost, and reported SOC density, we finally estimate the potential area of global permafrost thaw, and associated organic carbon in a warmer-than-present climate. Our assessment of global permafrost stability highlights that alpine permafrost and the associated carbon pool could be disproportionately important in determining the global permafrost-climate feedback.

## Results and discussion

### Stratigraphy and age model of the Kunlun Pass Section

The KP site is located on the northern Tibetan Plateau (35°39′N, 94°03′E, elevation ~4.7 km) south of the Eastern Kunlun Mountains that separate the modern cold steppe environments (Supplementary Fig. 1a) from the hyper-arid desert climate of the Qaidam Basin to the north^25^ (Fig. 1b). Modern glaciers are well-developed in the surrounding mountain ranges^25^. The sequence of the KP section is dominated by laminated dark grey calcareous mudstone interbedded with sparse greyish siltstone and sandstone (Supplementary Fig. 1b–d), that have been interpreted as deep-lake deposits^26,27^. This deep lake shrank and finally vanished in response to regional climate change during Late Cenozoic aridification of Asia^28^, with subsequent exhumation of the Eastern Kunlun Shan tilting the Plio-Pleistocene sequence of the KP section and facilitating field sampling^27^.

As shown on the thin sections (Supplementary Fig. 2), both endogenic carbonate (micrite) and allogenic (detrital) carbonates occur in the KP deposits. Detrital carbonate inputs are generally >200 μm while micrites are smaller (<200 μm) in grain size. Detrital carbonate inputs in the KP section are mainly derived from the Permian-Triassic carbonate-rich basement rocks in the surrounding mountain belts (Fig. 1), with low δ^18^O_c_ values −11 to −14‰, VPDB^29^. Detrital carbonate fragments come from basement rocks that have undergone thermal events at higher than surface temperature, thus, they should show higher clumped isotope (Δ_47_) temperatures and more negative δ^18^O_c_ values if they recrystallised in the presence of water of similar δ^18^O_w_ composition to surface waters^30^. Based on the correlation between the δ^18^O_c_ and Δ_47_-derived temperature, we developed an approach to minimise the potential effect of detrital carbonates contamination on carbonate isotopic results (carbonate δ^18^O_c_, δ^13^C_c_, Δ_47_) (see “Methods”; Supplementary Fig. 3).

Published magnetostratigraphy^27^ and biostratigraphy^26^ enabled the development of an age model using piecewise linear interpolation (Supplementary Fig. 4). The depositional age of the strata in the section span from 4.3 to 0.8 Ma. Spectral analysis of carbon and oxygen isotopes, as well as carbonate content records using the untuned age model resolved eccentricity, obliquity, and precession-scale variations (Supplementary Fig. 5), giving us confidence in the age estimates.

### Multi-proxy framework utilised

Carbonate δ^18^O_c_ has been widely used as an indicator of the variability in the precipitation/evaporation balance of lakes^18^, while carbonate δ^13^C_c_ provides important information on the composition of dissolved inorganic carbon (DIC) and reflects biologic productivity as well as mixing of DIC from different sources (i.e., lake waters, groundwater, and the atmosphere)^31^. For a closed lake basin system like the KP site, carbonate δ^18^O_c_ and δ^13^C_c_ usually show strong covariance (*r* > 0.7)^19^, while the sediment CaCO_3_ content should reflect changes in lake water CO_2_ due to biological activity (photosynthesis, respiration, and production of organic matter) or temperature^32^. The concentration of TOC in sediments should reflect both autochthonous and allochthonous sources^33^. C/N ratios of organic matter in lake sediments are widely used to distinguish between land-plant and aquatic origins of sedimentary organic matter: algae and cyanobacteria typically have C/N ratios between 4 and 10 whereas vascular land plants have C/N ratios of over 20^34^, though this ratio can be modified somewhat by decomposition^35^. When aquatic productivity is the dominant source of organic matter in a closed lake basin, increases in sediment TOC and TN, as well as a decrease in C/N, imply an increase in primary productivity probably associated with climate warming^36^. The δ^13^C_org_ from organic matter in lake sediments can also indicate the source of organic matter and climatic conditions^34^. Recent studies of Tibetan alpine lakes show that measured δ^13^C_org_ values from lake sediments can be related to the distribution of aquatic macrophytes, which are sensitive to water depth^37^. Submerged macrophytes with high δ^13^C_org_ values are dominant in shallow lakes where light penetrates to the lakebed, whereas algae with relatively low δ^13^C_org_ values dominate in deep lake environments, driving a negative correlation between δ^13^C_org_ and lake level for lakes with primarily autochthonous (internally produced) organic matter^37^. At the same time, during cooler periods, enhanced glacial growth accelerates erosion, resulting in an increase in the grain size of sediments in areas of sediment deposition^20^. In conjunction with the aforementioned proxies that are directly or indirectly associated with temperature, the Δ_47_ composition of endogenic carbonates from lake sediments provides quantitative constraints on lake water temperatures, which can, in turn, be used to derive MAAT^38^.

### Qualitative proxies for paleoenvironmental changes

**Fig. 2:**
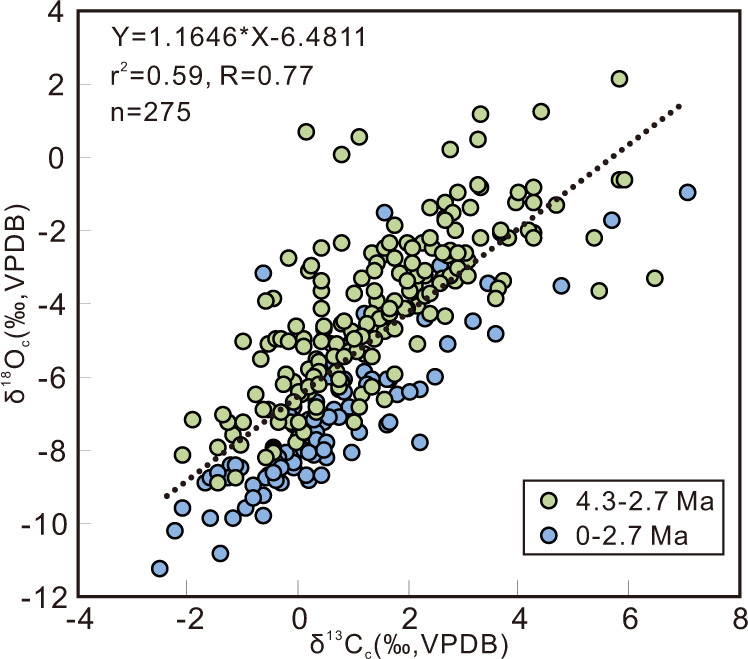
A comparison of oxygen and carbon isotope data from the Kunlun Pass section. The positive correlation (*R* > 0.7) between the parameters is typical of a closed lacustrine depositional environment for this set of strata^19^.

**Fig. 3:**
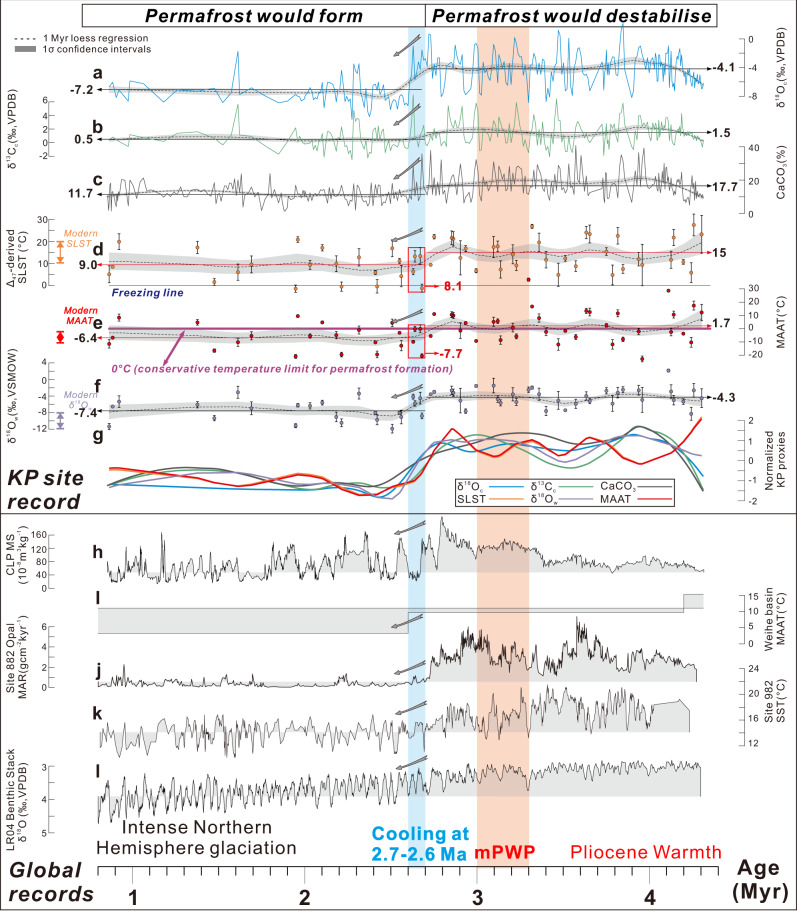
Paleoclimate records of the Kunlun Pass (KP) section, northern Tibetan Plateau, and global proxies. **a** Lacustrine δ^18^O_c_ record from the KP section. **b** Lacustrine δ^13^C_c_ record from the KP site. **c** Carbonate content from the KP section. **d** Surface lake summer temperature (SLST) from the KP section based on clumped isotope thermometry. **e** Mean annual air temperature (MAAT) from the KP section estimated by applying a transfer function relating SLST and MAAT^45^. **f** δ^18^O_w (SMOW)_ record from the KP section. The dashed line in **a**–**f** is the bootstrap plot (1 Myr loess regression) derived from each detailed record. The error bars in **d**–**f** represent the 1σ error of SLST, MAAT, and δ^18^O_w_ of each sample. **g** Normalised plots showing trends in paleoclimate proxy records from the KP section. These trends are bootstrap plots (1 Myr loess regression) derived from detailed records shown in (**a**–**f**). **h** Magnetic susceptibility (MS) record from the Chinese Loess Plateau (CLP)^28^, showing aridification at 2.7–2.6 Ma due to intensified winter monsoon associated with the global cooling. **i** MAAT record from the Weihe basin^46^. **j** Biogenic opal mass accumulation rates (MAR) at ODP Site 882^16^. **k** Sea Surface Temperature reconstruction for ODP Site 982^47^. **l** Global benthic δ^18^O stack^17^. Note that the cooling event at 2.7–2.6 Ma on the northern Tibetan Plateau is simultaneous with the intensification of Northern Hemisphere Glaciation (blue shaded area). The orange shaded area demarcates the mPWP (3.3–3.0 Ma). Modern MAAT, SLST, and meteoric water δ^18^O_w (SMOW)_ values at the KP section from previous work are shown^25^. Calculation of mean values and error bars (1σ) are in Supplementary Data 1. The numbers marked in **a**–**g** represent the mean value of each proxy during the interval 4.3–2.7 Ma and 2.7–0 Ma, respectively.

There is a positive correlation (*r* > 0.7) between oxygen and carbon isotopic compositions of bulk carbonate throughout the whole section (Fig. 2) that supports the sedimentary interpretation of a closed lacustrine depositional environment for these strata^19^, and in closed basins, changes in evaporation and precipitation balance related to lake level variation likely drive variation in δ^18^O_c_ and δ^13^C_c_^18^. Statistical analyses (see “Methods”) demonstrate that there is evidence for a step change at 2.7 Ma in all qualitative and quantitative proxy records for the KP site succession. For example, δ^18^O_c_, δ^13^C_c_, and CaCO_3_ content all shift from consistently high values between 4.3 and 2.7 Ma to low values after 2.7 Ma (Fig. 3a–c).

For all the samples from 4.3 to 0.8 Ma, the mean C/N ratio is 5.6 ± 2.3 (*n* = 334), while >95% of C/N ratios range between 2.6 and 10, indicating a predominately algal origin for organic matter^34^ (Supplementary Fig. 6a). A few samples (less than 5% of the total) with elevated TOC content also have a high C/N ratio (>10), suggesting only a minor contribution of terrestrial vegetation to organic matter in lake sediments. The higher TOC and TN values before 2.7 Ma are consistent with higher algal productivity associated with a warmer climate at the KP site during the Pliocene (Supplementary Fig. 6b, c). Both C/N ratios and δ^13^C_org_ fluctuate independent of stratigraphic boundaries, indicating that the degradation of organic matter during early diagenesis had only a minor influence on δ^13^C_org_ and C/N ratios (Supplementary Fig. 6a, 6d). The mean grain size record also oscillates and exhibits lower values between 4.3 and 2.7 Ma and high values after 2.7 Ma (Supplementary Fig. 6e).

Given that the high covariance of δ^18^O_c_ and δ^13^C_c_ throughout the section, the increased mean grain size of the sediments, and published data indicating regional aridification during the Plio-Pleistocene climate transition^28^, we infer that cooling at the KP site at 2.7 Ma, rather than opening of the hydrological system (e.g., lake level rise overtopping the topographic basin), provides a better explanation of the observations. Cooling at 2.7 Ma would have reduced lake evaporation resulting in a lowering of δ^18^O_c_, δ^13^C_c_, and CaCO_3_ content values (Fig. 3a–c). Lower lake temperature would have reduced primary productivity and driven further shifts of δ^13^C_c_, CaCO_3_ content, TN, and TOC values to lower values (Fig. 3b, c; Supplementary Fig. 6b, c). This regional cooling coincident with the intensification of Northern Hemisphere glaciation would also have driven the development of the glaciers in the northern Tibetan Plateau^39^ and enhanced glacial erosion in the surrounding mountain ranges. The increased input of coarser-grained sediments (Supplementary Fig. 6e) to the KP sites would have led to the lake likely shrinking, consistent with the observed increase in δ^13^C_org_ (Supplementary Fig. 6d) that has recently been used as a lake level indicator for alpine lakes in the northern Tibetan Plateau^37^. Further, the divergence between δ^13^C_c_ and δ^13^C_org_ at 2.7 Ma suggests that δ^13^C_org_ record may not always be positively correlated with productivity in closed-lake systems that are dominated by autochthonous organic matter sources. Changes in the proportion of aquatic and submerged plants with different δ^13^C_org_ values could play an important role in controlling variations in the bulk δ^13^C_org_ of lake sediments. In concert, these qualitative proxies support regional cooling at 2.7 Ma coincident with the intensification of Northern Hemisphere glaciation that in turn, played a fundamental role in changing paleoenvironments at the KP site, including by influencing evaporation rates, impacting lake area and productivity, and sedimentation.

### Quantitative temperature constraints indicate stepped cooling since the Pliocene

Recent clumped isotope studies indicate that lacustrine carbonates reflect warm-season near-surface water temperature^18,38,40,41^, and therefore we interpreted Δ_47_-derived temperatures as a proxy for summer lake surface temperatures (SLST). We would expect millennial and orbital-scale climate variability was a feature of both Pleistocene and Pliocene terrestrial paleoclimate records, as with marine records, but with variations being much larger in amplitude and proxy records noisier in terrestrial environments, particularly high-elevation localities such as the KP site. We also would predict that all SLST should be above 0 °C, because otherwise, the data would imply permanently frozen (or possibly hypersaline) lakes, where carbonate precipitation should be inhibited. We note that given the error bounds on the data, all SLST values are consistent with temperature above freezing (Fig. 3d).

The SLST reconstruction exhibits substantial, stepwise changes over the last 4.3 Ma (Fig. 3d), with temperatures fluctuating between 4.8 ± 4.5 (1σ) and 27.5° ± 4.9 °C from 4.3 to 2.7 Ma and varying between −1.4 ± 1.6 and 20.8 ± 1.3 °C between 2.7 and 0.8 Ma. The uncertainty includes both analytical error and the propagated error when converting the Δ_47_ values to the temperatures using the temperature-∆_47_ calibration of Bernasconi, Müller^42^ (see “Methods ”and Supplementary Data 1). Mean SLST values are 15.0 ± 6.9 °C (1σ) between 4.3 and 2.7 Ma, decreasing rapidly to 8.1 ± 6.9 °C at 2.7–2.6 Ma with a mean value of 9.0 ± 6.8 °C between 2.7 and 0.8 Ma (Fig. 2d). The mean Pliocene SLST of ~15 °C in the KP section based on Δ_47_ is consistent with the ca. 10–17 °C temperature estimates from aquatic plants, ostracods, and mollusk shells^43,44^.

**Fig. 4:**
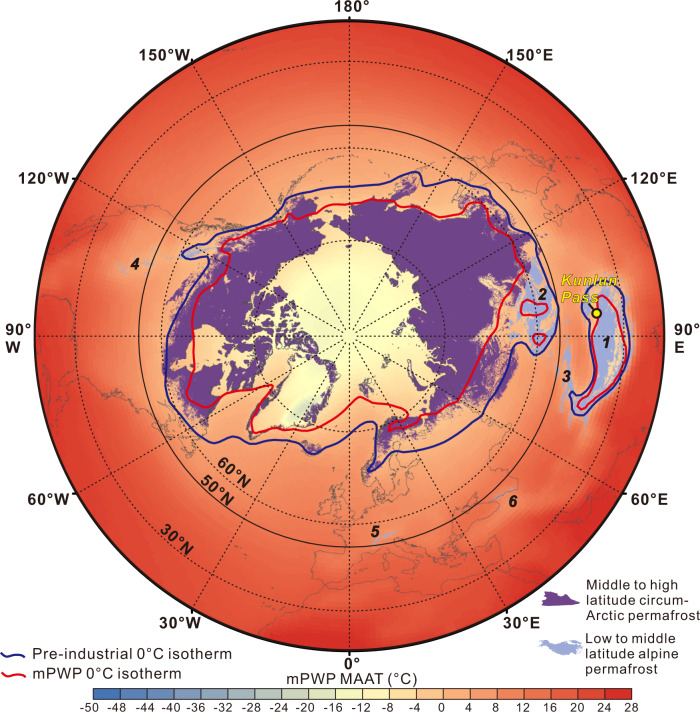
Simulated mean annual air temperature (MAAT) during the mid-Pliocene Warm Period (mPWP). **a** Pliocene Model Intercomparison Project Phase 2 (PlioMIP2)-based climate model simulation^68^, showing MAAT and the 0 °C isotherm during the mPWP and pre-industrial period, respectively. Region marked with 1-6 refers to the Tibetan Plateau-Pamir, Altai Mountains-Mongolia-Yablonoi-Sayan, Tian Shan, Rocky Mountains, Alps, and Caucasus, respectively.

Using a published relationship between SLST and MAAT^45^, we estimate that local MAAT from 4.3 to 0.8 Ma is ~13–17 °C cooler than SLST (Fig. 3e). The MAAT reconstruction consequently shows the same stepwise changes over the last 4.3 Ma (Fig. 3e), fluctuating between −12.2 ± 3.7 (1σ) and 17.5° ± 3.3 °C from 4.3 to 2.7 Ma and varying between −20.1 ± 1.4 (1σ) and 9.5 ± 0.9 °C between 2.7 and 0.8 Ma (uncertainty includes analytical error, propagated error when transferring the Δ_47_ values to the temperatures using the temperature-∆_47_ calibration of Bernasconi, Müller^42^ and the error when transferring the SLST to MAAT; see “Methods” and Supplementary Data 1). Mean MAAT values average 1.7 °C from 4.3 to 2.7 Ma, and decreased at 2.7–2.6 Ma to mean values of −6.4 °C, and further decreased to −7.7 °C at 2.7–0.8 Ma (Fig. 3e), similar to the local modern MAAT (−6 to −7 °C)^25,44^. These results imply >0 °C MAAT on the northern Tibetan Plateau during the Pliocene, which is consistent with PlioMIP2 climate simulations (Fig. 4). The Δ_47_–based estimate of local MAAT during the Pliocene of >0 °C is also broadly compatible with previous estimates of 10 ± 8 °C derived using the δ^18^O_w_ calculated from carbonate in bone and paleo-water^25^. The 2.7 Ma climate cooling is well recorded in records from across the Northern Hemisphere^15–17,28,46,47^.

δ^18^O_w_ values were calculated for each sample using Δ_47_-derived SLST estimates, measured δ^18^O_c_, and a published calibration^48^ (Fig. 3f). Reconstructed lake water δ^18^O_w_ has a mean value of −4.3 ± 1.8‰ from 4.3 to 2.7 Ma, and decreased to −7.4 ± 2.6‰ at 2.7–2.6 M that partly overlaps with the value of local modern meteoric waters (−11.9 to −7.7‰)^25^ (Fig. 3f). Evaporative enrichment of ^18^O in a closed lake system results in elevated δ^18^O_w_ values^19^. The decrease in lake water δ^18^O_w_ value at the KP site at 2.7–2.6 Ma may be associated with reduced evaporation in this region.

Collectively, multiple proxies indicate paleoclimate change at 2.7–2.6 Ma at the KP site in the northern Tibetan Plateau, likely related to regional cooling that was coincident with the intensification of the Northern Hemisphere Glaciation (NHG) (Fig. 3h–j)^16^. An ~8.1 °C decrease in local MAAT at 2.7–2.6 Ma is derived from the Δ_47_ paleothermometer and transfer function that relates late surface temperature to MAAT.

### Global permafrost stability and affected carbon

Global permafrost is mainly distributed in circumarctic and alpine regions, with the Tibetan Plateau being the largest alpine permafrost region on the Earth (Fig. 1a). The presence and stability of permafrost are fundamentally sensitive to changes in energy balance^2,6^. Assuming that our Earth would evolve to a mPWP-like climate in the near future, permafrost regions that exceed the temperature threshold for permafrost persistence will eventually thaw, exposing soil carbon to decomposition and lateral export. Thus, understanding the sensitivity of the carbon stocks in both circumarctic and alpine permafrost regions in a warmer-than-present climate is of critical importance regionally and globally.

To explore potential implications of paleo and near-future climate change of permafrost climate feedbacks, we combined our paleoclimate reconstruction with mPWP climate model results to assess global permafrost stability and affected carbon in a warmer-than-present climatic condition. Specifically, for both circumarctic and alpine permafrost regions, we assessed the distribution of permafrost, permafrost carbon storage, and potential permafrost-climate feedbacks under a mPWP-like climate. Because surface vegetation, moisture, and soil parameters can substantially affect the propagation of surface energy into the subsurface^22,23^, the precise MAAT of permafrost formation and stability can vary from several degrees below zero to a few degrees above depending on local conditions^22,23^. To determine which modern MAAT best represent the modern distribution of the permafrost region, we compare multiple modern MAAT isotherms (i.e., −2, −1.5, −1, −0.5, and 0 °C) with the modern distribution of the global permafrost region (Supplementary Fig. 7). At a global scale, the modern 0 °C MAAT isotherm best matches the distribution of permafrost (see “Methods”), and we thus use 0 °C as the conservative temperature threshold for global permafrost formation and persistence. While not perfect, this value is also widely used to model the modern permafrost area^49^.

Figure 4 shows a map of simulated mPWP global MAAT and the location of simulated mPWP and pre-industrial 0 °C MAAT isotherms. In the alpine Tibetan region, the mPWP 0 °C isotherm is in the centre of the plateau, while the pre-industrial 0 °C isotherm is situated at the margin of the plateau (Fig. 4). Outside the Tibetan Plateau, the mPWP 0 °C MAAT isotherm is generally located above 60°N with a few exceptions in North America and Eurasia, while the pre-industrial 0 °C MAAT isotherm is generally located above 50°N. From the mPWP to the pre-industrial period, the 0 °C isotherm on the Tibetan Plateau expands outwards, nearly doubling its area, while the 0 °C MAAT isotherm in the middle- to high-altitude migrates southwards by ~10° on average (Fig. 3a).

**Fig. 5:**
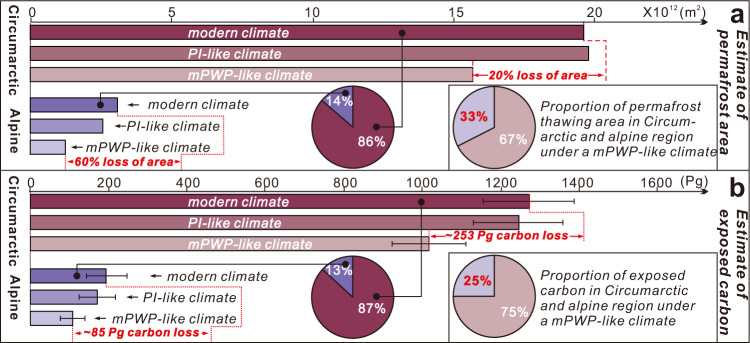
Estimated permafrost thawing and carbon affected under warmer climatic conditions. **a** Estimates of permafrost destabilisation and associated organic carbon from circumarctic and alpine permafrost regions in mPWP-like and pre-industrial(PI)-like climatic conditions. In a mPWP-like climate, around ~20% of circumarctic permafrost (containing ~253 Pg of carbon) would cross the 0 °C isotherm compared with ~60% of alpine permafrost (containing ~85 Pg of carbon). **b** Proportion of carbon in the circumarctic and alpine permafrost regions in modern climate and the proportion of carbon in the circumarctic and alpine permafrost regions in a mPWP-like climate (assuming total mineralisation of thawed carbon). Note that although the total amount of carbon in the alpine permafrost region is ~13% of total carbon trapped in modern permafrost, the ~85 Pg of carbon exposed to thaw from alpine permafrost represents ~25% of the total exposed carbon from permafrost in a mPWP-like climate. The error bars shown in b represent the 1σ error of amount of permafrost carbon with the calculation details shown in Supplementary Data 3. Region marked with 1–6 refers to the Tibetan Plateau-Pamir, Altai Mountains-Mongolia-Yablonoi-Sayan, Tian Shan, Rocky Mountains, Alps, and Caucasus, respectively.

Using this 0 °C MAAT isotherm as a conservative limit of permafrost persistence, the northern and southern edges of the Tibetan Plateau would become unstable under a mPWP-like climate. The estimated low to middle alpine permafrost regions in this study mainly consist of Tibetan Plateau-Pamir and Altai Mountains-Mongolia-Yablonoi-Sayan in addition to Tian Shan, Rocky Mountains, Alps, and Caucasus (Fig. 4). Using the present-day-permafrost distribution^1^ and the simulated mPWP 0 °C MAAT isotherm (Fig. 4), we find that about ~20% of the circumarctic permafrost would be destabilised (~3.9 × 10^12^ m^2^), but ~60% of alpine permafrost (~1.9 × 10^12^ m^2^) would be destabilised (Fig. 4). Using carbon estimates from modern permafrost regions^2,3^, we calculate the total carbon potentially affected under a future climate scenario similar to that of the mPWP (see Calculation of permafrost thawing area and carbon release in the “Methods” section). In our scenario of a mPWP-like climate, ~254 ± 23 Pg of organic carbon is potentially affected in the circumarctic permafrost zone and ~85 ± 60 Pg of organic carbon is affected in the alpine permafrost zone (Fig. 5).

### Glaciation and cooling on the Tibetan Plateau

A decrease in local MAAT in the Tibetan Plateau and adjacent regions during the Late Cenozoic is usually linked with global cooling and/or surface uplift^46,50^. Notably, the timing of 8.1 ± 7.5 °C decreases in MAAT inferred from Δ_47_ at the KP site coincides with a change in global radiative forcing based on reconstructions of atmospheric CO_2_^51^. A 2.7–2.6 Ma cooling event is recorded widely in the Northern Hemisphere in multiple proxy records. This feature is observed in the Lake El’gygytgyn record from the Arctic^15^, magnetic susceptibility data from the Chinese Loess Plateau^28^ (Fig. 3h), phytoliths in the Weihe basin in central China^46^ (Fig. 3i), the North Pacific biogenic opal records^16^ (Fig. 3j), North Pacific sea surface temperature^47^ (Fig. 3k), and benthic foraminiferal δ^18^O^17^ (Fig. 3l). Therefore, the cooling indicated by the Δ_47_ record likely reflects global cooling during the intensification of glaciation. Monitoring and simulations of climate change reveal that high-elevation regions are warming relatively faster than adjacent areas at lower elevation across the globe^52^. Lower elevation (<500 m) reconstructions from a similar latitude (~34 °N) derived from phytoliths in the Weihe basin of central China^46^ (Fig. 3i) indicated a decrease in MAAT of ~6 °C at 4–2 Ma^46^ (Fig. 3i). The larger temperature change in the high altitude Kunlun Pass of 8.1 ± 7.5 °C, compared to the Weihe basin, could be due to high-elevation temperature amplification^52^ (i.e., larger temperature changes at high-elevations in comparison with changes at low-elevations during global temperature change), although the difference may not be statistically significant.

To assess the possibility that surface uplift in the northern Tibetan Plateau occurred at ~2.7 Ma, we provide a first-order estimate that teases out the global temperature change effect on the local MAAT in the northern Tibetan Plateau, based on our knowledge of the correlation between MAAT variation in the northern Tibetan Plateau and global MAAT change in recent years due to anthropogenic greenhouse gas emissions. The last 50 years of temperature monitoring records a ~2.0 °C MAAT increase at high elevation on the northern Tibetan Plateau and a ~1.3 °C global temperature increase^52–54^, with a linear correlation between warming of high-altitude temperatures on the northern Tibetan Plateau and global temperatures (Supplementary Fig. 8). According to the transfer function established from this correction (see “Methods”), a global decrease in temperature of ~2–4 °C since 2.7 Ma^9,13,14,24,53^ should locally be expressed as a 7.8 ± 1.5 °C MAAT decrease on the northern Tibetan Plateau, which is within the error of 8.1 ± 7.5 °C observed cooling at 2.7–2.6 Ma at the KP site. Given the 4–5 °C/km modern lapse rate on the northern Tibetan Plateau^55^, this ~0.3 °C difference reflects approximately 0.1 km of local surface uplift (See calculation is in Supplementary Data 1). We thus infer that although minor surface uplift of the northern Tibetan Plateau cannot be ruled out for some of the observed cooling at the KP site at 2.7–2.6 Ma, most of the record reflects the regional expression of global climate change with high-elevation temperature amplification.

We also compare results with other published clumped isotope data and with PlioMIP2 mPWP model simulations. Our MAAT reconstruction from the KP section indicates an average MAAT of 1.7 °C in the northern Tibetan Plateau from 4.3 to 2.7 Ma (Fig. 3f), while in the southern Tibetan Plateau, clumped isotope analysis based on aquatic gastropods in the Zhada basin suggest a MAAT > 0 °C during the Pliocene^50^. Our MAAT reconstruction from the KP section indicates an average MAAT of ~0 °C at the KP site during the mPWP (Fig. 3f; Supplementary Data 1). This is consistent with the PlioMIP2 mPWP MAAT simulation result which shows that the 0 °C MAAT isotherm line during the mPWP goes by the KP site (Fig. 4). These consistencies between geological records from local studied sites and global mPWP MAAT simulation results not only give us confidence in both temperature proxy results and the simulations, but also provide insights on using the mPWP 0 °C MAAT isotherm simulation to explore the stability of modern permafrost in the near future (see “Methods”).

### Surprising vulnerability and importance of alpine permafrost carbon

Though alpine permafrost regions only contain 14% of organic matter stocks in the global permafrost zone, our calculations, albeit for a simplified analog-based warming scenario, suggest they could play an outsized role in determining the permafrost climate feedback. Enhanced thermal vulnerability of alpine permafrost regions has been supported by the monitoring of global MAAT, which shows greater warming in low-latitude, high-elevation regions compared with high-latitude circumarctic regions^52^. Given the current 400+ ppmv CO_2_ level in the atmosphere (equivalent to ~800 Pg of carbon)^9^, the mPWP-based results indicate a large quantity of carbon could be thawed in circumarctic and alpine permafrost regions. Because of its climate vulnerability, alpine permafrost region could disproportionately account for ~25% (i.e., ~85 ± 60 Pg) of the carbon affected by a transition from modern to mPWP-like climate conditions (Fig. 3c).

We recognise that there is not a well-established relationship between amount of permafrost thaw and amount of greenhouse gas release^11^, and that the distribution of modern permafrost is influenced by the legacy of the last ice ages^56,57^. Although these factors hamper a precise calculation of how much carbon will be released when from thawing permafrost, our simple scenario allows projection of which permafrost-containing regions are likely to become unstable under climatic conditions similar to the mPWP. As regional MAAT in areas of permafrost storage exceed the temperature threshold for permafrost persistence, the total carbon that is stored in the permafrost soil column will eventually thaw and destabilise organic matter, with microbial production of greenhouse gases that could be taken up by biota and/or ultimately released into the atmosphere. Ultimately, the amount of CO_2_, CH_4_, and N_2_O released to the atmosphere from these regions will depend on specific ecological conditions, including slope, hydrological status, duration of seasonal thaw, disturbance, and amount of gradual versus abrupt thaw^2,10^. However, upland areas—including on the Tibetan Plateau—are often more vulnerable to carbon release following permafrost degradation because of aerobic respiration and thermo-erosion^10^, suggesting that alpine permafrost carbon might be dually responsive to climate change. This implies that detailed long-term monitoring of alpine regions is urgently needed to quantify organic matter stocks, lateral transport (e.g., fluvial and colluvial processes), and vertical greenhouse gas flux. Constraining alpine temperature amplification and carbon cycle dynamics under different global warming scenarios will be critical to determining the amount of additional warming that this carbon release from permafrost could cause.

## Methods

### Kunlun Pass site and age model

The KP section is the same section that was studied previously for magnetostratigraphy with an approximately two-metre resolution^27^ and multiple faunal layers by Li, Xie^26^. Given problems in determining reliable magnetostratigraphy within conglomerate layers with sand-sized matrix, we focused on fine-grained lacustrine deposits in the middle to the upper parts of the section and re-correlate the observed polarities using the Geomagnetic Polarity Time Scale (GPTS)^58^. The coarse yellowish sandstone in the upper parts of the section and the black organic-rich mudstone layer that preserve leaves enable us to correlate our newly measured section to a previous study^27^. We correlate two short normal intervals of N2 and N3 to chrons C1r.1n and C1r.2n, respectively. The long normal interval N4 can be correlated to the C2An.1n-C2An.3n. Four short normal intervals of N5, N6, N7, and N8 to chrons C3n.1n, C3n.2n, C3n.3n, and C3r.4n, respectively. The age tie points used to establish the age model for the section are given in Supplementary Fig. 4. We thus determined the age model by piecewise linear interpolation.

To confirm the age model (4.3–0.8 Ma), a total of 344 samples were collected for carbonate (CaCO_3_) content, δ^18^O_c_, and δ^13^C_c_ measurement (Fig. 3a–c). The obvious fluctuations in all three records indicate potential orbital cycles (Fig. 2a–c). If our magnetostratigraphy-based age model is corrected, we should resolve orbital cycles from this record. We used these high-resolution records (Supplementary Fig. 3a–c) to carry out time-frequency analysis. Power spectral analysis and Fast Fourier transformation (FFT) were performed using *Acycle* software^59^. Using the untuned age model, spectral analysis on CaCO_3_ content, carbon, and oxygen isotope records resolves orbital cycles, indicating eccentricity, obliquity, and precession (Supplementary Fig. 5b, c), giving us confidence in the age model. To better explore the paleoclimate change during the Plio-Pleistocene climate transition, the sampling rate of CaCO_3_ content and isotope samples is higher (~12 kyr) for the time interval from 4.3 to 1.8 Ma. For the time interval between 1.8 and 0.8 Ma, the sampling rate of CaCO_3_ content varies between ~12 and ~20 kyr while the sampling rate of isotope samples ranges from ~40 to ~130 kyr (Supplementary Fig. 5).

### Sample measurements

Approximately 50 g of freeze-dried sediment from each specimen was homogenised for carbonate δ^18^O_c_ and δ ^13^C_c_, clumped isotope (Δ_47_), carbonate content (CaCO_3_), organic δ^13^C_org_, TOC and TN measurements.

#### Carbonate δ^18^O_c_ and δ^13^C_c_ measurements

δ^18^O_c_ and ^13^C_c_ of carbonate samples were analysed at the Stable Isotope Ratios in the Environment Analytical Laboratory, University of Rochester. Results are reported in per mil (‰), relative to the Vienna PeeDee Belemnite (VPDB) standard. Around 0.3 g of each sample that display no visible veins or recrystallisation were sieved through a 200-mesh screen, homogenised and subsequently reacted with 50 ml 3% H_2_O_2_ for 5 h to remove organic matter. Each sample was then rinsed with deionized water. After drying at room temperature (~20 °C), the powder was weighed, sealed in a glass vial, and flushed with helium gas. The powder was then reacted with pure phosphoric acid at 70 °C to release CO_2_ gas. Analyses were performed using a Thermo Delta plus XP CF-IRMS with a GasBench II peripheral device. To calculate isotopic ratios, 3 in-house standards calibrated to international standards (namely NBS-18, NBS-19, and L-SVEC) were used. Analytical errors for δ^18^O and δ^13^C are within ±0.1‰ (1σ) and ±0.06‰ (1σ), respectively. A total of 275 δ^18^O_c_ and δ^13^C_c_ values were obtained.

#### Clumped isotope (Δ_47_) measurements

Clumped isotope analyses were carried out in the Tripati Lab at the University of California–Los Angeles, following the methods of Upadhyay, Lucarelli^60^. A total of 57 samples were analysed. During the pretreatment, approximately 0.3 g of each sample that displays no visible veins or recrystallisation was sieved through a 70-mesh screen, homogenised and subsequently reacted with 50 ml 3% H_2_O_2_ for 5 h to remove organic matter. Each sample was then rinsed with deionized water and dried at 40 °C for 24 h. Samples were then ground and homogenised using an agate mortar and pestle. Before analysing on a Thermo 253 Gas Source isotope ratio mass spectrometer, three to seven aliquots of each sample were reacted at 90 °C with 103% phosphoric acid for ~20 min. The resultant CO_2_ was cryogenically purified. To normalise sample Δ_47_ values, equilibrium CO_2_ standard gases prepared at 1000 and 25 °C were analysed concurrently with the sample unknowns. Clumped isotope data were reported relative to the absolute reference frame (ARF) of Dennis, Affek^61^. An acid fractionation factor of 0.082‰ at 90 °C was used for Δ_47_ corrections^62^. Seven internal carbonate standards (CMTile, Carmel Chalk, Veinstrom, and ETH 1, ETH 2, ETH 3, ETH 4) were analysed multiple times for calibration (2-3 carbonate standards were analysed every 5–7 samples; with >500 carbonate standards are analysed) for calibration. We used the software “Easotope” for data processing^63^. The isotopic abundance ratios (including ^17^O value) used were those recommended by Daëron, Blamart^64^. During the course of analysis, the mean Δ_47_ values and standard deviation (SD) from these eight standards were 0.371‰ ± 0.029‰ (*n* = 66), 0.666‰ ± 0.024‰ (*n* = 77), 0.714‰ ± 0.026‰ (*n* = 78), 0.259‰ ± 0.020‰ (*n* = 74), 0.275‰ ± 0.064‰ (*n* = 66), 0.690‰ ± 0.021‰ (*n* = 72), and 0.530‰ ± 0.057‰ (*n* = 67), respectively. Carbonate formation temperatures were calculated from the ∆_47_ results using the temperature-∆_47_ calibration of Bernasconi, Müller^42^ (Supplementary Data 1). Using a different calibration slightly shifts temperatures^40,65^, but the MAAT reconstruction still indicates a ~8 °C cooling at 2.7–2.6 Ma with an average of >0 °C MAAT from 4.3 to 2.7 Ma and an average of less than 0 °C MAAT since 2.7 Ma (Supplementary Data 2). These MAAT reconstructions still support our inference of permafrost-free environment in the northern Tibetan Plateau during the Pliocene and subsequent formation of permafrost since 2.7 Ma. Thus, the conclusions of the study remain the same, regardless of the calibration that is used. The temperature-∆_47_ calibration of ^40^, Petersen, Defliese^65^ is given in the Supplementary Data 2 for comparison. The MAAT uncertainty includes both analytical error and the propagated error when transferring the Δ_47_ values to the temperatures using the temperature-∆_47_ calibration of Bernasconi, Müller^42^ (Supplementary Data 1).

#### Carbonate content measurements

The bulk carbonate content was determined at State Key Laboratory of Loess and Quaternary Geology, Institute of Earth Environment, Chinese Academy of Sciences, following the neutralisation-titration method of Hesse^66^. A total of 344 data were obtained. Approximately 0.3 g of material from each sample was decarbonated with 30 ml HCl (0.2 mol/L) for 24 h. Then, 10 ml centrifuged supernatant was titrated with NaOH (0.1 mol/L) with two drops of phenolphthalein solution as the neutralisation indicator. In parallel, a corresponding blank experiment was carried out. The content of carbonate is calculated as follows:

$${{{{{\rm{CaCO}}}}}_{3}}\,{{{{{\rm{content}}}}}}=[150\times {C_{1}}-15\times ({V_{1}}-{V_{2}})\,\times {C2}]/{A},$$

where *C*_*1*_ and *C*_*2*_ are the concentration of the HCl and NaOH solution, respectively; and *V*_*1*_ and *V*_*2*_ are the volumes of the NaOH solution used for titration of the sample and the blank, respectively. *A* refers to the amount of each sediment material. The analytical precision of the CaCO_3_ content is ±0.5%.

#### Organic δ^13^C_org_ (organic carbon isotopic composition) measurements

The organic δ^13^C_org_ measurements were determined at State Key Laboratory of Loess and Quaternary Geology, Institute of Earth Environment of Chinese Academy of Sciences. A total of 344 values were obtained. The samples were sieved through a 200-mesh screen, homogenised, and then treated with 2 M HCL for 24 h at room temperature to remove carbonates. The samples were subsequently rinsed to a pH of approximately 7 with deionized water and dried at 40 °C. Each sample was then combusted for 4 h at 850 °C in evacuated sealed quartz tubes in the presence of 1 g of CuO, 1 g of Cu, and Pt foil. The CO_2_ gas was then cryogenically purified. The isotopic ratios of the purified CO_2_ gas were measured using a Finnigan MAT gas source mass spectrometer and reported using δ-notation as per mil (‰) differences relative to the V-PDB scale for carbon. The precision of the δ^13^C analyses was smaller than 0.2‰.

#### TOC and TN measurements

TOC and TN measurements were made at Qinghai Institute of Salt Lakes, Chinese Academy of Sciences. A total of 344 data were obtained. Approximately 0.5 g of each sample was ground using an agate mortar and pestle, sieved through a 200-mesh screen, and homogenised. Each sample was then treated with 2 M HCl for 24 h at room temperature to remove carbonate. Subsequently, the samples were rinsed to a pH of approximately 7 with deionized water and dried at 40 °C for 24 h. The concentrations of total TOC and TN in the samples were determined using an elemental analyser (vario EL cube). The analytical error for both the TOC and TN content is ±0.1%.

#### Grain size measurements

Grain size measurements were determined at State Key Laboratory of Loess and Quaternary Geology, IEECAS. A total of 344 samples were analysed. During the pretreatment, 10% HCl and 10% H_2_O_2_ were used to remove organic matter and carbonates in the samples. All the samples were then put with deionized water and kept overnight in beakers. Before grain size analysis, water was siphoned off and each sample was then dispersed in an ultrasonic bath in a 10 ml vial in a 10% (NaPO_3_)_6_ solution for 10 minutes. Finally, grain size distributions were obtained using a Malvern 2000 laser instrument.

### Statistical analyses confirm a climate change event at 2.7 Ma at the KP site

All δ^18^O_c_, δ^13^C_c_, CaCO_3_ content records shifted to a lower value at 2.7 Ma. We note this shift at 2.7 Ma is noisy in KP site SLST and MAAT records. To evaluate if there is robust evidence for a shift in regional climate at 2.7 Ma, we conducted a Student’s T-test. Using the δ^18^O_c_, δ^13^C_c_, CaCO_3_ content, TOC, TN, δ^13^C_org_, grain size, SLST, MAAT, and δ^18^O_w_ records. This method determines the probability that two populations are the same with respect to the variable tested. The fundamental criterion of the Student’s T-test is the *p* value: if the *p* value is less than 0.05, there is a >95% level of confidence that the two age distributions are not the same. Lower *p* values provide a higher probability of rejecting the null hypothesis that there is no difference between the two populations.

We divided each record using multiple age boundaries (i.e., 2.7, 1.0, 1.5, 2.0, 2.5, 3.0, 3.5, and 4.0 Ma) and conducted a Student’s T-test. This was done with δ^18^O_c_, δ^13^C_c_, CaCO_3_ content, TOC, TN, δ^13^C_org_, grain size, SLST, MAAT, and δ^18^O_w_ records. The Student’s T-test results, shown in Supplementary Table 1, reveal the *p* values derived from all records are smallest if 2.7 Ma is the time when there is a shift, indicating that the 2.7 Ma boundary is robust.

Using 1 million years as an age window, we also used the bootstrap plots (loess regression) to evaluate the trends in δ^18^O_c_, δ^13^C_c_, CaCO_3_ content, TOC, TN, δ^13^C_org_, grain size, SLST, MAAT, and δ^18^O_w_ record, respectively (Fig. 3; Supplementary Fig. 6). The normalised plots also show a shift at 2.7 Ma (Fig. 3g), consistent with a change in regional climate at 2.7 Ma at the KP site.

Collectively, our statistical analyses confirm that a climate change event at 2.7 Ma is recorded in multiple proxy records from the KP section, with the direction of change consistent with cooling. This inference of cooling is observed in other proxy records from across Eurasia and the Northern Hemisphere^15–17,28,46,47^ (Fig. 3h–l).

### Sample petrography, carbonate facies description, and diagenetic screening

All KP section samples were examined under reflected light using a microscope. Fifteen representative samples were impregnated with epoxy (to hold them together) and prepared as thin sections. These sections were examined using cross-polarised light microscopy at the University of Rochester to evaluate the origin and potential for diagenesis of carbonate. Thirteen of the fifteen samples are predominantly fine-grained (clay to silt-sized), containing micrite, clay, and quartz (Supplementary Fig. 2a). We also examined 2 moderately sorted, very fine sandstones that show quartz, detrital carbonate, and metamorphic grains, with a clay- to silt-sized matrix (Supplementary Fig. 2b, c). The minor amount of quartz and detrital carbonates grains are derived from locally exposed basement rocks including carbonate outcrops in the surrounding regions^29^. Carbonate in over 90% of samples is dominantly micritic (Supplementary Fig. 2a–c). Minor blocky sparite or microspar in pore spaces was observed in the samples, and likely accounts for the minimal lithification of the sediment. The lack of lithification and dominantly micritic texture of the carbonates demonstrates little to no recrystallisation associated with diagenesis (Supplementary Fig. 2a). We interpret the minor sparite associated with pore spaces as early diagenesis associated with primary fluid in the sediment. This inference is further supported by the shallow depth of burial of sediments (<300 m).

### Methods of removing detrital carbonate component

We developed a new approach to reduce the effect of detrital carbonates contamination on carbonate isotopic data. Two test samples (16KL102 and 16KL152) were sieved through a 230-mesh screen and a 70-mesh screen, respectively. Each test sample was divided into four groups (non-sieved, <212 μm, 212–63 μm, <63 μm) depending on grain size. Materials from each group were then homogenised and subsequently reacted with 50 ml 3% H_2_O_2_ for 5 h to remove organic matter. Materials from each group were rinsed with deionized water and dried at 40 °C for 24 h, then ground and homogenised in an agate mortar. Two to three replicates from each group were weighed and then analysed on a Thermo 253 Gas Source isotope ratio mass spectrometer. Measurement and normalisation of these samples followed the procedure outlined in the Clumped Isotope (Δ_47_) Measurements section of this paper, stated above.

A total of 16 replicates for sample 16KL152 and 13 replicates for sample 16KL102 were analysed and results are displayed in Supplementary Fig. 3. The non-sieved group, which included detrital grains, yields a lower δ^18^O_c_ value but a higher Δ_47_-derived temperature than from the <212 μm, the 212–63 μm, and the <63 μm groups. In addition, the ranges of δ^18^O_c_ value and Δ_47_-derived temperature for <212 μm, 212–63 μm, and <63 μm groups are similar. We thus infer that the <212 μm sized mesh removed a portion of detrital carbonate fragments in the samples. For each sample, two groups of replicates can be identified in the δ^18^O_c_ vs Δ_47_-derived temperature cross plot (Supplementary Fig. 3): a first group of replicates with high temperatures (*T* = 20–95 °C for sample 16KL102; *T* = 25–60 °C for sample 16KL152) and low δ^18^O_c_ values (δ^18^O_c_ = −9.0 to −8.0‰ for sample 16KL102; δ^18^O_c_ = −6.3 to −5.7‰ for sample 16KL152), and a second group with low temperatures (*T* = 5–20 °C for sample 16KL102; *T* = 5–25 °C for sample 16KL152) and high δ^18^O_c_ values (δ^18^O_c_ = −7.7 to −7.4‰ for sample 16KL102; δ^18^O_c_ = −5.5 to −5.3‰ for sample 16KL152). The first group mainly consists of non-sieved samples while the second group corresponds to sieved samples (including <212 μm, 212–63 μm, and <63 μm). We infer that the first group of replicates corresponds with the detrital carbonate component in the sample, while the second group of replicates corresponds with the authigenic carbonate in the sample (Supplementary Fig. 3).

According to these observations, we first used 70-mesh screen to remove a portion of detrital carbonates in all the samples and subsequently conducted δ^18^O_c_ and δ^13^C_c_, clumped isotope (Δ_47_) measurement. We then construct a δ^18^O_c_ vs Δ_47_-derived temperature cross plot for replicates of each sample. We infer that those replicates with higher δ^18^O_c_ values and lower Δ_47_-derived temperatures reflects authigenic carbonate with less detrital carbonate component. Among 57 samples for Δ_47_ isotopic measurement, 37 samples display a positive correlation between their δ^18^O_c_ and the Δ_47_ value (Supplementary Data 1). For each sample, three replicates on the “authigenic carbonate end-member” (i.e., with the lower Δ_47_-derived temperature and the higher δ^18^O_c_ value) are used to calculate their mean and error on δ^18^O_c_, δ^13^C_c_, and Δ_47_-derived temperature (Supplementary Data 1) and calculated for their mean and error of δ^18^O_c_, δ^13^C_c_, and Δ_47_-derived temperature. The 1σ error of δ^13^C_c_ and δ^18^O_c_ is ≤0.3‰, while their 1σ error of the Δ_47_-derived temperature is between 0.9 °C and 4.8 °C. Eight exceptions (samples 16KL20, 160KL106, 160KL222, 160KL236, 160KL248, 160KL266, and 160KL293) contain only two replicates due to a lack of replicates toward the “authigenic carbonate end”. The 1σ error of δ^13^C_c_ and δ^18^O_c_ of these eight samples is smaller than 0.2‰ and 0.6‰, respectively, while their 1σ error of the Δ_47_-derived temperature is between 0.9 and 8.5 °C. Our approach of removing detrital carbonates and data screening give us a maximum SLST. For the remaining 20 samples that do not show a positive correlation between δ^18^O_c_ and Δ_47_ values, we statistically constrain the three replicates whose 1σ error of Δ_47_-derived temperature and the 1σ error of δ^18^O_c_ are the smallest (i.e., most reproducible). For example, regarding each sample, we first list all the combinations of 3 different replicates. We then calculate the 1σ error of δ^18^O_c_ and Δ_47_ of all these combinations. We use the combination with three replicates whose 1σ error of Δ_47_-derived temperature and the 1σ error of δ^18^O_c_ are the smallest at the same time to represent the results of this sample. For these 20 samples, their 1σ error of Δ_47_ ranges from 0.4 to 7.7 °C while 1σ error of δ^13^C_c_ and δ^18^O_c_ is ≤0.3‰, respectively. Two samples (16KL160 and 16KL331) yield 45.6 ± 2.2 °C (1σ) and 37.5 ± 0.8 °C (1σ), respectively. Given that the modern global surface lake summer temperature is below 30 °C^45^, the high Δ_47_-derived temperatures of these two samples likely may reflect detrital carbonate inputs. We thus do not consider these two samples for the MAAT estimate. The raw data screening process and plots are given in Supplementary Data 1.

### Correlation between global temperature and regional MAAT changes

We evaluate the potential correlation between changes in global temperature and MAAT in the northern Tibetan Plateau using temperature information from weather stations. Using the global temperature variations reported by Morice, Kennedy^54^ and the MAAT on the northern Tibetan Plateau reported by Wang, Fan^53^, we made a cross plot of anomalies in global temperature and MAAT in the northern Tibetan Plateau for the 1961–2010 period (both defined relative to the 1961–1990 average). This plot shows a strong linear correlation (*r* = 0.84) between these two anomaly data sets (Supplementary Fig. 8). A transfer function relating the global temperature variation to the MAAT variation in the northern Tibetan Plateau is established:

1 $${\Delta T}_{{{{{\rm{NTP}}}}}}=2.5725{{\times }}{\Delta T}_{{{{{\rm{G}}}}}}+0.0295$$

where Δ*T*_NTP_ is the variation of MAAT (°C) in the northern Tibetan Plateau in response to Δ*T*_G_ (global temperature variation, °C). This transfer function enables estimation of MAAT decrease in the northern Tibetan Plateau in response to a global decrease in temperature of ~2–4 °C at 2.7 Ma. We acknowledge that the relation between the Δ*T*_NTP_ and Δ*T*_G_ during the Plio-Pleistocene transition might not completely follow this transfer function. Despite uncertainties (e.g., continentality effect and moisture transport), this transfer function allows us to provide a first-order estimate of potential surface uplift in the northern Tibetan Plateau.

### Calculation of permafrost thawing area and corresponding carbon affected

According to definition, permafrost is ground with a temperature that remains at or below 0 °C for two or more years^4,6^. Thus, the permafrost persistence is basically temperature-dependent. Although more paleotemperature data derived from geological records are still needed, the consistencies between paleotemperature reconstruction from sediments in permafrost region (including KP site)^15,50^ and the simulations (Fig. 4), give us confidence in using the mPWP MAAT isotherm simulation results to explore the stability of modern permafrost across the globe in a warmer than today climate scenario in the near future.

To define the limit for the global permafrost persistence, we compare the modern permafrost area with multiple global modern and pre-industrial MAAT isotherms (i.e., −2, −1.5, −1.0, −0.5, and 0 °C). The −2, −1.5, −1.0, and −0.5 °C pre-industrial MAAT isotherm lines are located inside the extent of the modern permafrost region (Supplementary Fig. 7). This contradiction indicates these MAAT isotherms might not represent the threshold for global permafrost formation or persistence. On the other hand, the modern 0 °C MAAT isotherm better characterises the distribution of modern permafrost, and the pre-industrial 0 °C MAAT isotherm line generally defines the extent of the modern permafrost in different regions. We thus define 0 °C as the conservative temperature threshold for global permafrost persistence^4,6^. We recognise that this threshold overestimates permafrost in some regions and underestimates permafrost in others but it best represents the average distribution of permafrost and is also widely used in modelling the area of modern permafrost area^49^.

We also recognise that permafrost can also be destabilised due to other reasons, such as coastal erosion, infrastructure damage, landslides, ecosystem damage, etc. We believe a more complex specific modelling approach might help to gain realistic estimates; however, it is currently difficult to predict and even quantify all these parameters. Comprehensive quantitative impact of these unknown factors on the stability of global permafrost is worthy of further exploration and research. Despite uncertainties, our simple approach of defining 0 °C MAAT isotherm as the conservative temperature threshold for global permafrost enables us to provide a first-order quantitatively estimate of the areas that permafrost could be affected in a warmer than today climate scenario in the near future.

The present-day global permafrost distribution we used is based on a recent study from Obu, Westermann^49^. The middle to high latitude (between 50°N and 90°N) Arctic permafrost and low to middle latitude (between 25°N and 50°N) alpine permafrost regions (including the Tibetan Plateau- Pamir, Altai Mountains-Mongolia-Yablonoi-Sayan, Tian Shan, Rocky Mountains, Alps, and Caucasus.) are shown in both Figs. 1 and 4, with their area calculated using ArcGIS 10.1 (Supplementary Data 3).

The PlioMIP2-based climate simulation shown in Fig. 4 depicts the global mPWP MAAT with the mPWP and pre-industrial 0 °C MAAT isotherms. These two isotherms models the residual permafrost region in both middle to high latitude circumarctic permafrost and low to middle latitude alpine permafrost regions during the mPWP and pre-industrial periods, respectively. We loaded these two modelled 0 °C MAAT isotherm lines into ArcGIS 10.1 and calculated the circumarctic and alpine permafrost areas within these isotherms that correspond to mPWP-like and pre-industrial-like climates, respectively. Comparing the modelled permafrost areas in a mPWP-like warmer climate with the modern permafrost area allows us to estimate the potential area of permafrost thaw in the near future, under conditions similar to the mPWP. Comparing modelled permafrost areas in a pre-industrial-like climate with the modern permafrost area, allows us to estimate the amount of permafrost area that has thawed since the pre-industrial period (Supplementary Data 3).

To estimate SOCs stocks in these potential permafrost thaw regions, we multiplied the estimated permafrost thaw area by the reported mean SOC in these regions. We used SOC data from Hugelius, Strauss^4^ and used ~1300 Pg with an uncertainty range of ~1100 to 1500 Pg (mean SOC = 65 ± 6 kg/m^2^) to represent the total SOC stocks in the circumarctic permafrost region. We used the estimate by Mu, Zhang^3^ and used 160 ± 87 Pg to represent the total SOC stocks in the Tibetan Plateau and Pamir permafrost region. For other alpine permafrost thaw areas (including Pamir, Mongolia Altai Mountains-Mongolia-Yablonoi-Sayan, Tian Shan, Rocky Mountains, Alps, and Caucasus), we multiplied each permafrost thaw area by the corresponding mean SOC density to represent the total SOC stocks in these regions. The SOC for different regions was obtained from the compilation by Bockheim and Munroe^5^, who compiled many available SOC estimates. We use the maximum and minimum SOC value in each area to represent the uncertainty of the SOC value. We conducted Monte Carlo modelling assuming 1σ uncertainties that span the range corresponding SOC values, similar to methods used in estimations of circumarctic and alpine permafrost soil stocks^4^. The computation and mean value of SOC with 1σ error in the Tibetan Plateau-Pamir, Altai Mountains-Mongolia-Yaionoi-Sayan, Tian Shan, Rocky Mountains, Alps, Caucasus, and the Arctic permafrost region are given in Supplementary Data 3. Finally, after multiplying the permafrost thaw area by the corresponding SOC, the soil total carbon in the corresponding permafrost thaw region in a mPWP-like and pre-industrial-like climate are then calculated as the quantity of carbon that would be thawed in the near future and are shown in Fig. 4 and Supplementary Data 3.

### Climate simulation

Required spatial information for Pliocene and pre-industrial surface temperatures have been derived from the Pliocene Model Intercomparison Project Phase 2(PlioMIP2). PlioMIP2 co-ordinates climate modelling groups from around the world in completing climate simulations for the mPWP using a unified experimental design. Full details of the experimental design and boundary conditions used for PlioMIP2 modelling studies can be found in Haywood, Dowsett^67^ and Dowsett, Dolan^68^, respectively. In this study, we utilise the multi-model mean annual surface air temperature field from PlioMIP2 for both the mPWP (Eoi400 experiment) and pre-industrial era (E280 experiment; see Haywood, Tindall^69^). These data are used to plot the spatial position of the −2, −1.5, −1.0, −0.5, 0 °C MAAT isotherms. The estimated MAAT is an average of the 1 × 1° latitude/longitude grid box from each climate model.

## Supplementary information

Supplementary Information

Description of Additional Supplementary Files

Supplementary Data 1

Supplementary Data 2

Supplementary Data 3

## Data Availability

All new data produced for this study are from samples from a continuous Pliocene to Pleistocene lacustrine record on the northern Tibetan Plateau. The authors declare that all data supporting the findings of this study are available within the article and its Supplementary Data and are accessible online at 10.6084/m9.figshare.19033130.v2.

## Code availability

No code was developed for this study.

## References

[1] Brown, J., Ferrians, O., Heginbottom, J. A. & Melnikov, E. Circum-Arctic map of permafrost and ground-ice conditions, Version 2, 10.7265/skbg-kf16 (2002).

[2] Schuur EA (2015). Climate change and the permafrost carbon feedback. Nature.

[3] Mu C (2015). Organic carbon pools in permafrost regions on the Qinghai–Xizang (Tibetan) Plateau. Cryosphere.

[4] Hugelius G (2014). Estimated stocks of circumpolar permafrost carbon with quantified uncertainty ranges and identified data gaps. Biogeosciences.

[5] Bockheim JG, Munroe JS (2014). Organic carbon pools and genesis of alpine soils with permafrost: A review. Arct., Antarct., Alp. Res..

[6] Biskaborn BK (2019). Permafrost is warming at a global scale. Nat. Commun..

[7] Zimov SA, Schuur EAG, Chapin FS (2006). Permafrost and the Global Carbon Budget. Science.

[8] Koven CD (2011). Permafrost carbon-climate feedbacks accelerate global warming. Proc. Natl Acad. Sci. USA.

[9] IPCC. *Climate Change 2013: The Physical Science Basis*. *Contribution of Working Group I to the Fifth Assessment Report of the Intergovernmental Panel on Climate Change* 1535 (Cambridge University Press, 2013).

[10] Turetsky MR (2020). Carbon release through abrupt permafrost thaw. Nat. Geosci..

[11] McGuire AD (2018). Dependence of the evolution of carbon dynamics in the northern permafrost region on the trajectory of climate change. Proc. Natl Acad. Sci. USA.

[12] Abbott BW (2016). Biomass offsets little or none of permafrost carbon release from soils, streams, and wildfire: An expert assessment. Environ. Res. Lett..

[13] Burke K (2018). Pliocene and Eocene provide best analogs for near-future climates. Proc. Natl Acad. Sci. USA.

[14] Haywood AM, Dowsett HJ, Dolan AM (2016). Integrating geological archives and climate models for the mid-Pliocene warm period. Nat. Commun..

[15] Brigham-Grette J (2013). Pliocene warmth, polar amplification, and stepped Pleistocene cooling recorded in NE Arctic Russia. Science.

[16] Haug GH (2005). North Pacific seasonality and the glaciation of North America 2.7 million years ago. Nature.

[17] Lisiecki LE, Raymo ME (2005). A Pliocene‐Pleistocene stack of 57 globally distributed benthic δ^18^O records. Paleoceanography.

[18] Horton TW, Defliese WF, Tripati AK, Oze C (2016). Evaporation induced ^18^O and ^13^C enrichment in lake systems: A global perspective on hydrologic balance effects. Quat. Sci. Rev..

[19] Talbot M (1990). A review of the palaeohydrological interpretation of carbon and oxygen isotopic ratios in primary lacustrine carbonates. Chem. Geol.: Isot. Geosci. Sect..

[20] Leemann A, Niessen F (1994). Holocene glacial activity and climatic variations in the Swiss Alps: Reconstructing a continuous record from proglacial lake sediments. Holocene.

[21] Eiler JM (2011). Paleoclimate reconstruction using carbonate clumped isotope thermometry. Quat. Sci. Rev..

[22] Shur YL, Jorgenson MT (2007). Patterns of permafrost formation and degradation in relation to climate and ecosystems. Permafr. Periglac. Process..

[23] Loranty MM (2018). Reviews and syntheses: Changing ecosystem influences on soil thermal regimes in northern high-latitude permafrost regions. Biogeosciences.

[24] Haywood A (2013). Large-scale features of Pliocene climate: Results from the Pliocene Model Intercomparison Project. Climate.

[25] Wang Y (2008). Stable isotopes in fossil mammals, fish and shells from Kunlun Pass Basin, Tibetan Plateau: Paleo-climatic and paleo-elevation implications. Earth Planet. Sci. Lett..

[26] Li Q (2014). Vertebrate fossils on the roof of the world: Biostratigraphy and geochronology of high-elevation Kunlun Pass Basin, northern Tibetan Plateau, and basin history as related to the Kunlun strike-slip fault. Palaeogeogr., Palaeoclimatol., Palaeoecol..

[27] Song C (2005). Late Cenozoic high-resolution magnetostratigraphy in the Kunlun Pass Basin and its implications for the uplift of the northern Tibetan Plateau. Chin. Sci. Bull..

[28] Guo Z (2002). Onset of Asian desertification by 22 Myr ago inferred from loess deposits in China. Nature.

[29] Huang H, Di P, Chen D (2012). Sedimentary petrology and geochemistry of Permian-Triassic carbonate mounds in Long-Stone mountain in Bayan Har Basin. Acta Sedimentologica Sin..

[30] Garzione CN, Dettman DL, Horton BK (2004). Carbonate oxygen isotope paleoaltimetry: Evaluating the effect of diagenesis on paleoelevation estimates for the Tibetan plateau. Palaeogeogr., Palaeoclimatol., Palaeoecol..

[31] Hudson AM (2017). Stable C, O and clumped isotope systematics and 14C geochronology of carbonates from the Quaternary Chewaucan closed-basin lake system, Great Basin, USA: Implications for paleoenvironmental reconstructions using carbonates. Geochim. Cosmochim. Acta.

[32] Bozzano F, Marcoccia S, Barbieri M (1999). The role of calcium carbonate in the compressibility of Pliocene lacustrine deposits. Q. J. Eng. Geol. Hydrogeol..

[33] Meyers PA (2003). Applications of organic geochemistry to paleolimnological reconstructions: A summary of examples from the Laurentian Great Lakes. Org. Geochem..

[34] Meyers PA (1994). Preservation of elemental and isotopic source identification of sedimentary organic matter. Chem. Geol..

[35] Abbott BW (2016). Using multi-tracer inference to move beyond single-catchment ecohydrology. Earth-Sci. Rev..

[36] Nara F (2005). Response of phytoplankton productivity to climate change recorded by sedimentary photosynthetic pigments in Lake Hovsgol (Mongolia) for the last 23,000 years. Quat. Int..

[37] Li X, Liu W, Xu L (2019). Evaluation of lacustrine organic δ^13^C as a lake-level indicator: A case study of Lake Qinghai and the satellite lakes on the Tibetan Plateau. Palaeogeogr., Palaeoclimatol., Palaeoecol..

[38] Huntington K, Wernicke B, Eiler J (2010). Influence of climate change and uplift on Colorado Plateau paleotemperatures from carbonate clumped isotope thermometry. Tectonics.

[39] Nie J, Pullen A, Garzione CN, Peng W, Wang Z (2018). Pre-Quaternary decoupling between Asian aridification and high dust accumulation rates. Sci. Adv..

[40] Li H (2021). Mass 47 clumped isotope signatures in modern lacustrine authigenic carbonates in Western China and other regions and implications for paleotemperature and paleoelevation reconstructions. Earth Planet. Sci. Lett..

[41] Hren MT (2013). Terrestrial cooling in Northern Europe during the Eocene–Oligocene transition. Proc. Natl Acad. Sci. USA.

[42] Bernasconi SM (2018). Reducing uncertainties in carbonate clumped isotope analysis through consistent carbonate-based standardization. Geochem. Geophys. Geosyst..

[43] Wu Y (2001). Quaternary geomorphological evolution of the Kunlun Pass area and uplift of the Qinghai-Xizang (Tibet) Plateau. Geomorphology.

[44] Pang, Q. *Contribution to the Geology of the Qinghai-Xizang (Tibet) Plateau* (ed The Editorial Committee on the Tibet Plateau Geological Papers) 151–165 (Geological Publishing house, 1982).

[45] Hren MT, Sheldon ND (2012). Temporal variations in lake water temperature: Paleoenvironmental implications of lake carbonate δ^18^O and temperature records. Earth Planet. Sci. Lett..

[46] Wang H (2019). Asian monsoon rainfall variation during the Pliocene forced by global temperature change. Nat. Commun..

[47] Herbert TD (2016). Late Miocene global cooling and the rise of modern ecosystems. Nat. Geosci..

[48] Kim S-T, O’Neil JR (1997). Equilibrium and nonequilibrium oxygen isotope effects in synthetic carbonates. Geochim. Cosmochim. Acta.

[49] Obu J (2019). Northern Hemisphere permafrost map based on TTOP modelling for 2000–2016 at 1 km^2^ scale. Earth-Sci. Rev..

[50] Huntington KW, Saylor J, Quade J, Hudson AM (2015). High late Miocene–Pliocene elevation of the Zhada Basin, southwestern Tibetan Plateau, from carbonate clumped isotope thermometry. GSA Bull..

[51] Bartoli G, Hönisch B, Zeebe RE (2011). Atmospheric CO_2_ decline during the Pliocene intensification of Northern Hemisphere glaciations. Paleoceanography.

[52] Wang Q, Fan X, Wang M (2016). Evidence of high-elevation amplification versus Arctic amplification. Sci. Rep..

[53] Wang Q, Fan X, Wang M (2014). Recent warming amplification over high elevation regions across the globe. Clim. Dyn..

[54] Morice CP, Kennedy JJ, Rayner NA, Jones PD (2012). Quantifying uncertainties in global and regional temperature change using an ensemble of observational estimates: The HadCRUT4 data set. J. Geophys. Res.: Atmospheres.

[55] Wang Y, Wang L, Li X, Chen D (2018). Temporal and spatial changes in estimated near-surface air temperature lapse rates on Tibetan Plateau. Int. J. Climatol..

[56] Lindgren A, Hugelius G, Kuhry P, Christensen TR, Vandenberghe J (2016). GIS-based maps and area estimates of northern hemisphere permafrost extent during the last glacial maximum. Permafr. Periglac. Process..

[57] Herzschuh U (2016). Glacial legacies on interglacial vegetation at the Pliocene-Pleistocene transition in NE Asia. Nat. Commun..

[58] Hilgen, F., Lourens, L. & Van Dam, J. *The Geologic Time Scale 2012* (eds Gradstein, F., Ogg, J., Schmitz, M. & Ogg, G.) 923–978 (Elsevier, 2012).

[59] Li M, Hinnov L, Kump L (2019). Acycle: Time-series analysis software for paleoclimate research and education. Comput. Geosci..

[60] Upadhyay D (2021). Carbonate clumped isotope analysis (Δ47) of 21 carbonate standards determined via gas-source isotope-ratio mass spectrometry on four instrumental configurations using carbonate-based standardization and multiyear data sets. Rapid Commun. Mass Spectrom..

[61] Dennis K, Affek H, Passey B, Schrag D, Eiler J (2011). Defining an absolute reference frame for ‘clumped’ isotope studies of CO_2_. Geochim. Cosmochim. Acta.

[62] Defliese WF, Hren MT, Lohmann KC (2015). Compositional and temperature effects of phosphoric acid fractionation on Δ_47_ analysis and implications for discrepant calibrations. Chem. Geol..

[63] John CM, Bowen D (2016). Community software for challenging isotope analysis: First applications of ‘Easotope’ to clumped isotopes. Rapid Commun. Mass Spectrom..

[64] Daëron M, Blamart D, Peral M, Affek HP (2016). Absolute isotopic abundance ratios and the accuracy of Δ47 measurements. Chem. Geol..

[65] Petersen SV (2019). Effects of improved ^17^O correction on interlaboratory agreement in clumped isotope calibrations, estimates of mineral-specific offsets, and temperature dependence of acid digestion fractionation. Geochem., Geophys., Geosyst..

[66] Hesse, P. *A Textbook of Soil Chemical Analysis* (Chemical Publishing Co., 1972).

[67] Haywood AM (2016). The Pliocene Model Intercomparison Project (PlioMIP) Phase 2: Scientific objectives and experimental design. Clim. Past.

[68] Dowsett H (2016). The PRISM4 (mid-Piacenzian) paleoenvironmental reconstruction. Clim. Past.

[69] Haywood A (2020). A return to large-scale features of Pliocene climate: The Pliocene Model Intercomparison Project Phase 2. Clim. Past.

[70] Fick SE, Hijmans RJ (2017). WorldClim 2: New 1-km spatial resolution climate surfaces for global land areas. Int. J. Climatol..

