# Supplementary material for: Alpine permafrost could account for a quarter of thawed carbon based on Plio-Pleistocene paleoclimate analogue
Source: Nat Commun. 2022 Mar 14;13:1329. doi: 10.1038/s41467-022-29011-2 (PMC8921200; doi:10.1038/s41467-022-29011-2)
Supplement: Supplementary file 1 — Supplementary Information [file 41467_2022_29011_MOESM1_ESM.pdf]

# Alpine permafrost could account for a quarter of thawed carbon based on Plio-Pleistocene paleoclimate analogue

## Supplementary Information

Feng Cheng[1,2], Carmala Garzione[2,3,4], Xiangzhong Li[5,6], Ulrich Salzmann[7], Florian Schwarz[7], Alan M. Haywood[8], Julia Tindall[8], Junsheng Nie[9], Lin Li[4], Lin Wang[10], Benjamin W. Abbott[11], Ben Elliott[12], Weiguo Liu[6], Deepshikha Upadhyay[12], Alexandrea Arnold[12], Aradhna Tripati[12]

[1] Key Laboratory of Orogenic Belts and Crustal Evolution, Ministry of Education, School of Earth and Space Sciences, Peking University, Beijing, 100871, China

[2] Department of Earth and Environmental Sciences, University of Rochester, Rochester, NY 14627, USA

[3] Department of Environmental Sciences, Rochester Institute of Technology, Rochester, NY 14623, USA

[4] College of Science, University of Arizona, Tucson, Arizona 85721, USA

[5] Yunnan Key Laboratory of Earth System Science, Yunnan University, Kunming, 650500, China

[6] State Key Laboratory of Loess and Quaternary Geology, Institute of Earth Environment, Chinese Academy of Science, Xi'an 710061, China

[7] Department of Geography and Environmental Sciences, Northumbria University, Newcastle upon Tyne, NE1 8ST, UK

[8] School of Earth and Environment, University of Leeds, Woodhouse Lane, Leeds, LS2 9JT, UK

[9] Key Laboratory of Western China's Environmental Systems (Ministry of Education), College of Earth and Environmental Sciences, Lanzhou University, Lanzhou 730000, China

[10] Department of Civil and Environmental Engineering, The Hong Kong University of Science and Technology, Hong Kong SAR, China

[11] Department of Plant and Wildlife Sciences, Brigham Young University, Provo, Utah, USA

[12] Department of Earth, Planetary, and Space Sciences, Department of Atmospheric and Oceanic Sciences, Institute of the Environment and Sustainability, Center for Diverse Leadership in Science, University of California, Los Angeles, CA 90095, USA

**Correspondence**: Feng Cheng (cfcf.chengfeng@gmail.com)

# Supplementary Figures 1-8; Supplementary Table 1

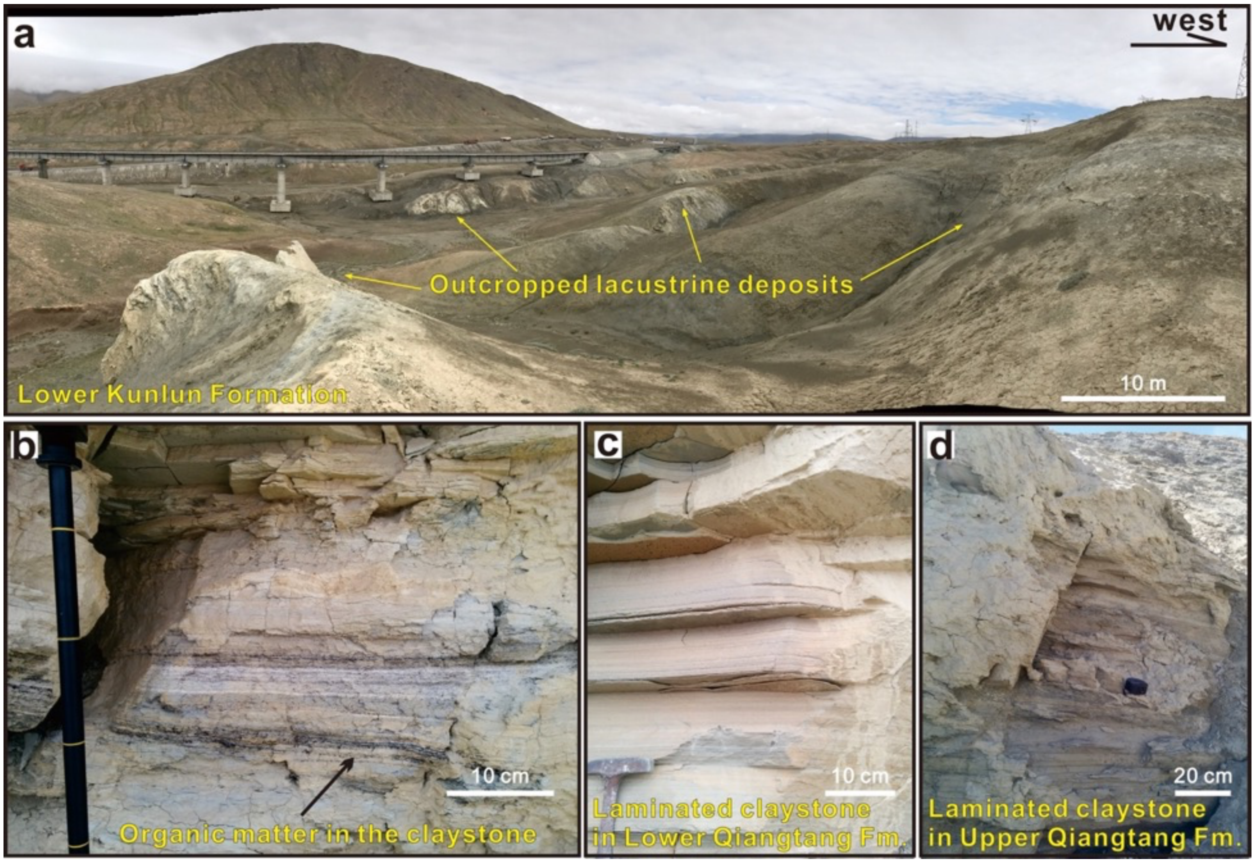


**Supplementary Figure 1. Photographs of Pliocene to Pleistocene strata outcropping in the Kunlun Pass Basin, northern Tibetan Plateau. a.** Image showing the present-day cold steppe environment and outcrops of lacustrine deposits in the KP section, northern Tibetan Plateau. **b**. Organic matter in claystone. **c**. Laminated claystone in the lower part of the KP section. **d**. Laminated claystone in the upper part of the KP section.

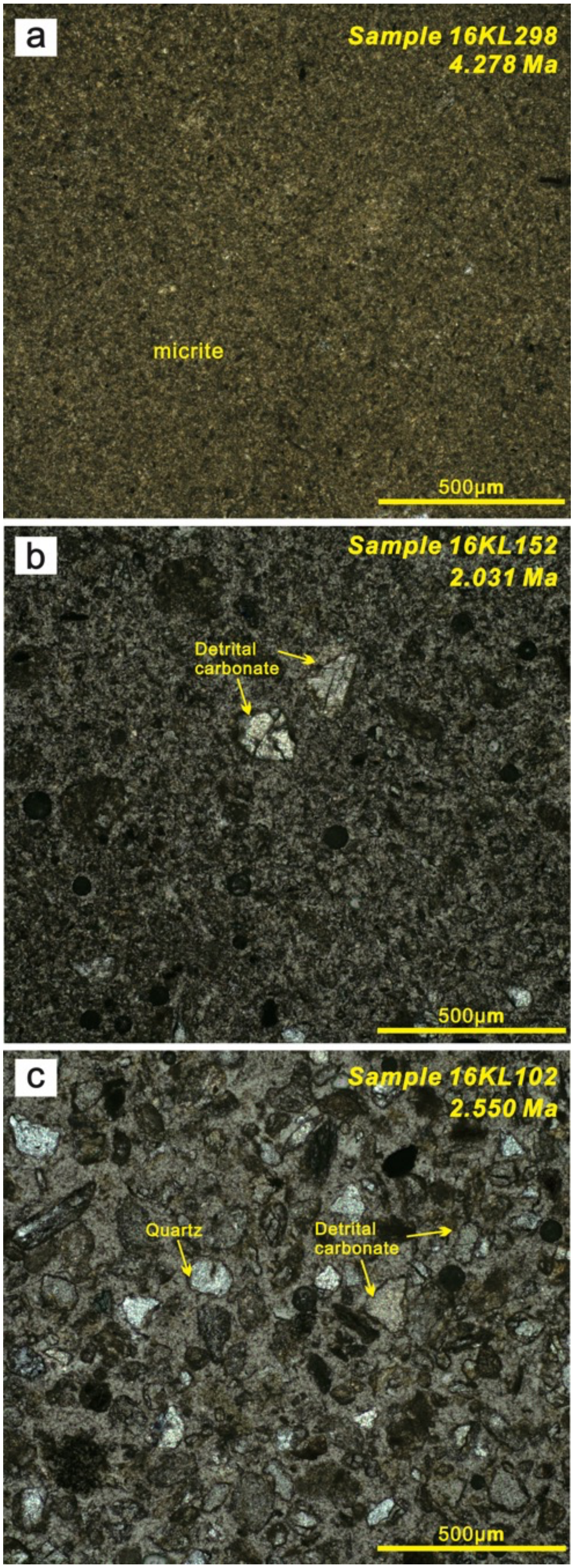


**Supplementary Figure 2. Images and Photomicrographs of representative thin sections from the KP section. a.** Cross-polarized light photomicrographs of laminated calcareous claystone samples (16KL298) in the KP section**.** Note that carbonate is dominantly micritic. Minor detrital carbonate inputs are observed in the sample. Minimal blocky sparry calcite or microspar around vugs were observed in the samples, indicating that samples were not impacted by diagenesis. **b**. Cross-polarized light photomicrograph of sample 16KL152 indicating detrital carbonates. **c**. Photomicrograph (under cross-polarized light) of sample 16KL102 indicating detrital carbonates and quartz. Detrital carbonate was largely removed through sample pre-treatment described in the Methods section.

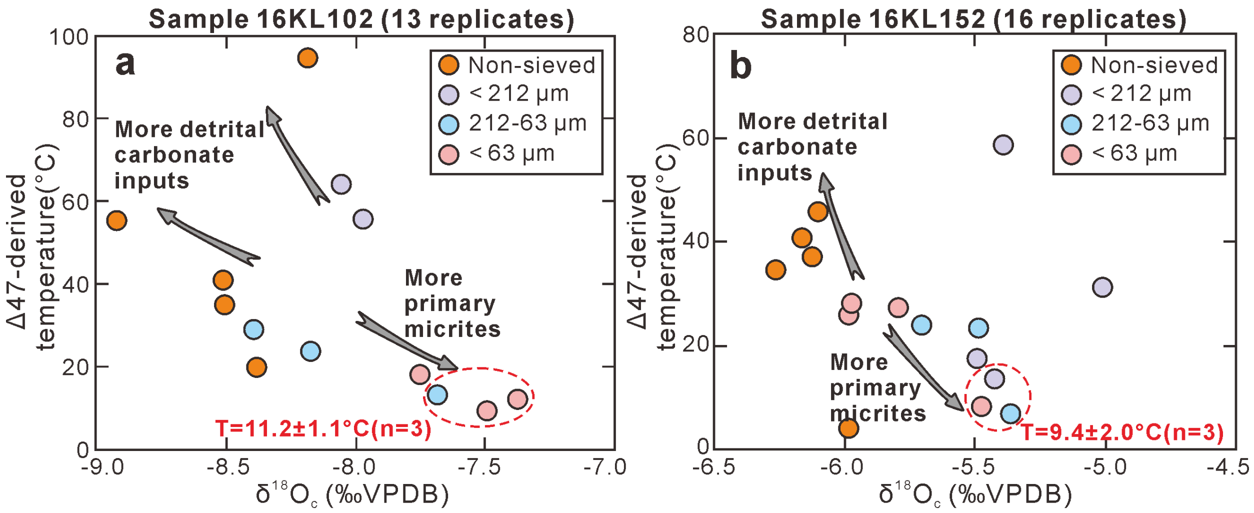


**Supplementary Figure 3. Clumped isotopes ($\Delta_{47}$) derived temperature and carbonate $\delta^{18}O_c$ cross plot. a**. cross plots for the sample 16KL102. **b**. cross plots for the sample 16KL102. The non-sieved group replicate yields a lower $\delta^{18}O_c$ value but a higher $\Delta_{47}$-derived temperature than replicates from the <212μm, the 212-63μm, and the <63μm groups. The range of $\delta^{18}O_c$ values and $\Delta_{47}$-derived temperatures for <212μm, 212-63μm and <63μm groups is similar. Two groups of replicates can be identified in the $\delta^{18}O_c$ vs $\Delta_{47}$-derived temperature cross plot: groups of replicates with high temperatures (T = 20-95 °C for sample 16KL102; T = 25-60 °C for sample 16KL152) and low $\delta^{18}O_c$ values ($\delta^{18}O_c$ = -9.0~-8.0‰ for sample 16KL102; $\delta^{18}O_c$ = -6.3~-5.7‰ for sample 16KL152), and a group with low temperatures (T = 5-20 °C for sample 16KL102; T = 5-25 °C for sample 16KL152) and high $\delta^{18}O_c$ values ($\delta^{18}O_c$ = -7.7~-7.4‰ for sample 16KL102; $\delta^{18}O_c$ = -5.5~-5.3‰ for sample 16KL152). Detrital carbonate fragments sourced from basement rocks that have undergone thermal events at higher than surface temperature should show higher clumped isotope ($\Delta_{47}$) temperatures and more negative $\delta^{18}O_c$ values if they recrystallised in the presence of water of similar $\delta^{18}O_w$ composition to surface waters[61]. We inferred that using 70-mesh screen removes detrital carbonates. The replicates with higher $\delta^{18}O_c$ values and lower $\Delta_{47}$-derived temperatures reflect authigenic carbonate with less detrital carbonate component.

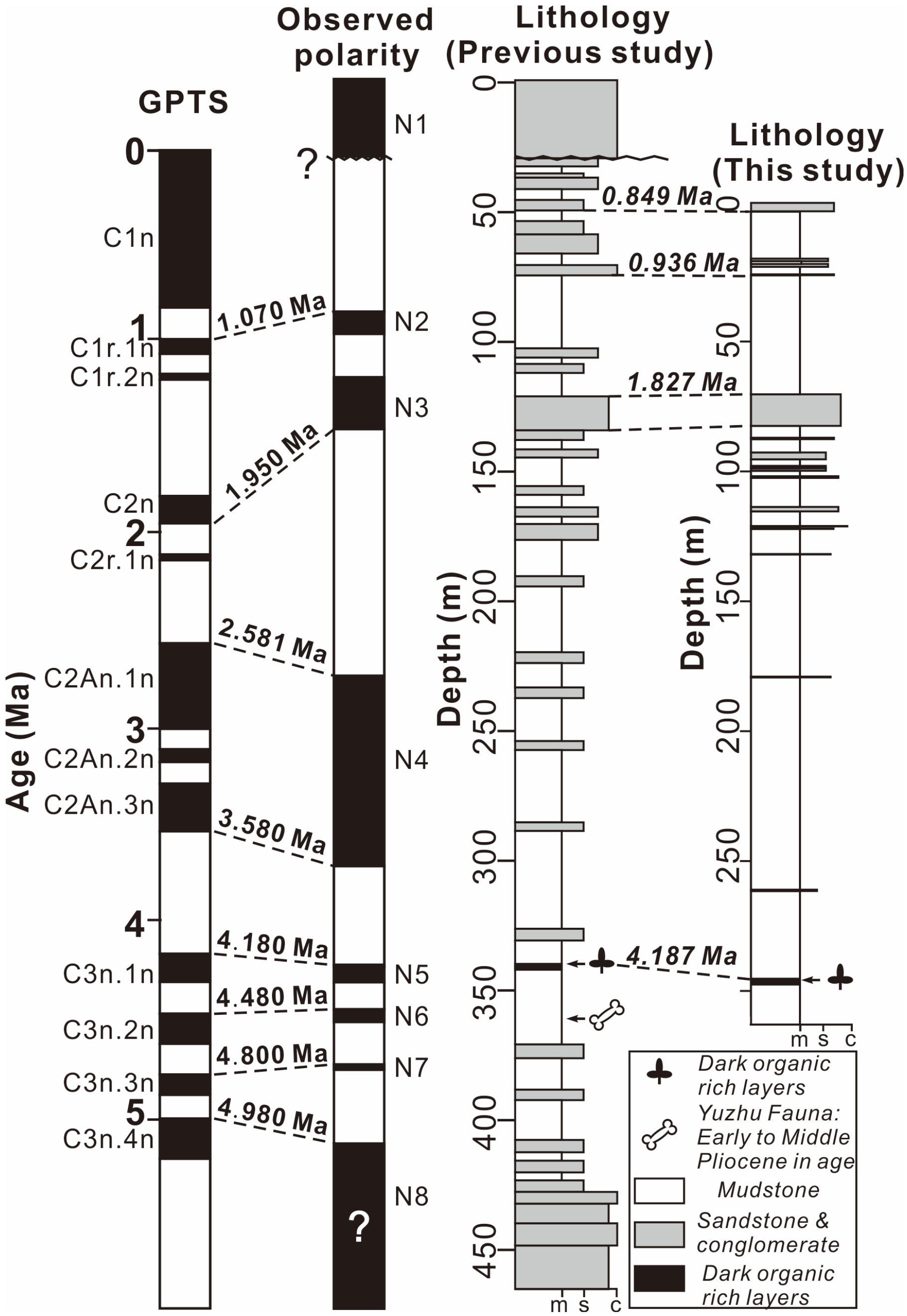


**Supplementary Figure 4. Age model and stratigraphic column of the KP section, northern Tibetan Plateau.** The magnetostratigraphic and biostratigraphic age constraints are from previous geological survey and magnetostratigraphy study[25, 26]. The difference in bar length in the column shows the variation of grain-size of the sediments at the KP section. Note that m, s, and c marked at the bottom o the column is abbreviation for mudstone, sandstone, and conglomerate, respectively.

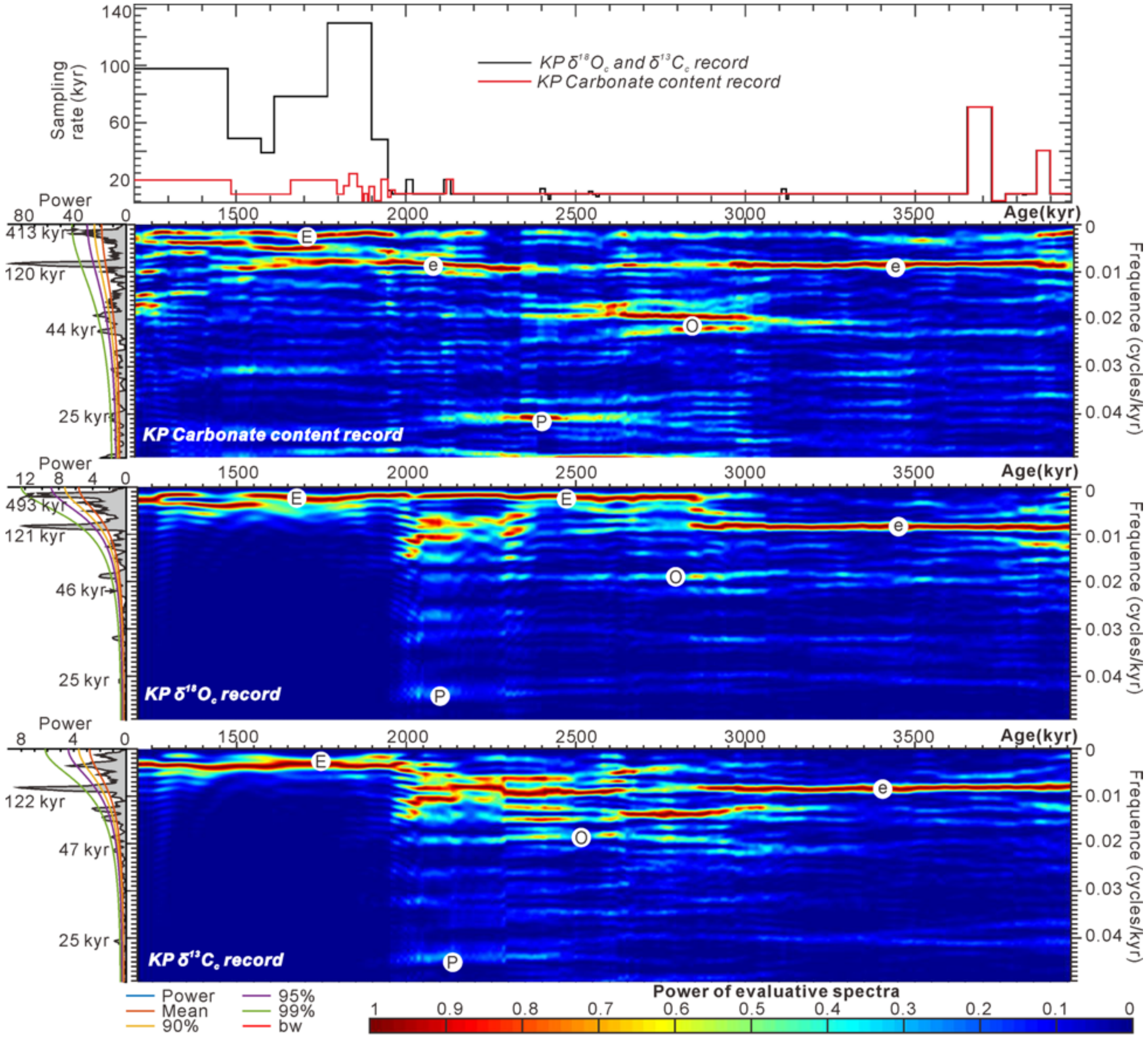


**Supplementary Figure 5. Sampling rate and cyclostratigraphy of the strata on the KP (Kunlun Pass) section**. Sampling interval for the $\delta^{18}O_c$, $\delta^{13}C_c$ and $CaCO_3$ content records from the KP site. b, c, d shows the FFT (Fast Fourier transform) spectrogram[68] of the untuned $CaCO_3$ content $\delta^{18}O_c$, and $\delta^{13}C_c$ series, respectively. Power spectrum analysis of the $2\pi$ multi-taper (MTM)[69] of the untuned $\delta^{18}O_c$, $\delta^{13}C_c$ and $CaCO_3$ content with robust AR(1) red-noise models[70] with a 20% median filter length and linear fitting. E, e, O and P in the figure refer to long eccentricity, short eccentricity, obliquity and precession, respectively. Note that spectral analysis using the untuned age model matches orbital cycles of eccentricity (~405 kyr, and ~100 kyr), obliquity (~41 kyr), and precession (~23 kyr) on carbon and oxygen isotope as well as $CaCO_3$ content.

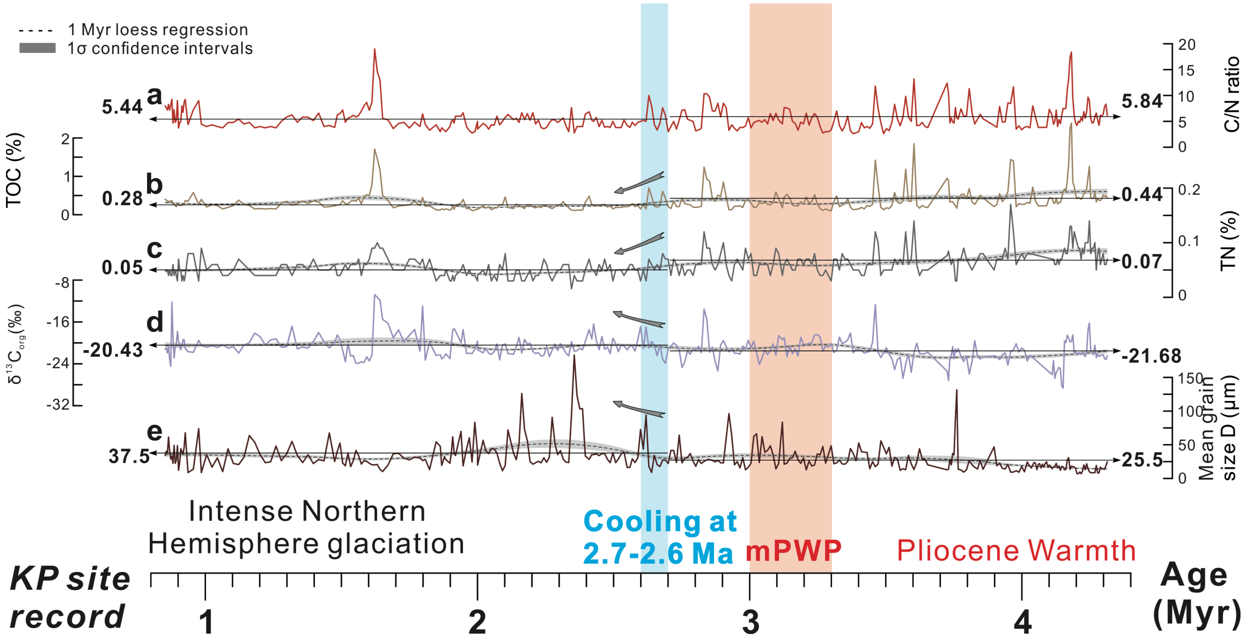


**Supplementary Figure 6. Paleoclimate records of the KP section, northern Tibetan Plateau**. a, C/N ratio (TOC/TN atomic ratio) record from the KP section (this study). b, TOC record from the KP section (this study). c, TN record from the KP section (this study). d, mean grain size D(4, 3) record from the KP section (this study). e, Organic $\delta^{13}C_{org}$ record from the KP section (this study). Note the low C/N ratio (less than 8 on average), potentially indicating predominately algal origin of the organic matter. The relative higher TN and TOC value prior to 2.7 Ma are consistent with a warmer climate at the studied site. The cooling at 2.7-2.6 Ma is hypothesized to have led to an increase in grain size (d) and a shrinking of the lake (e) in the KP section.

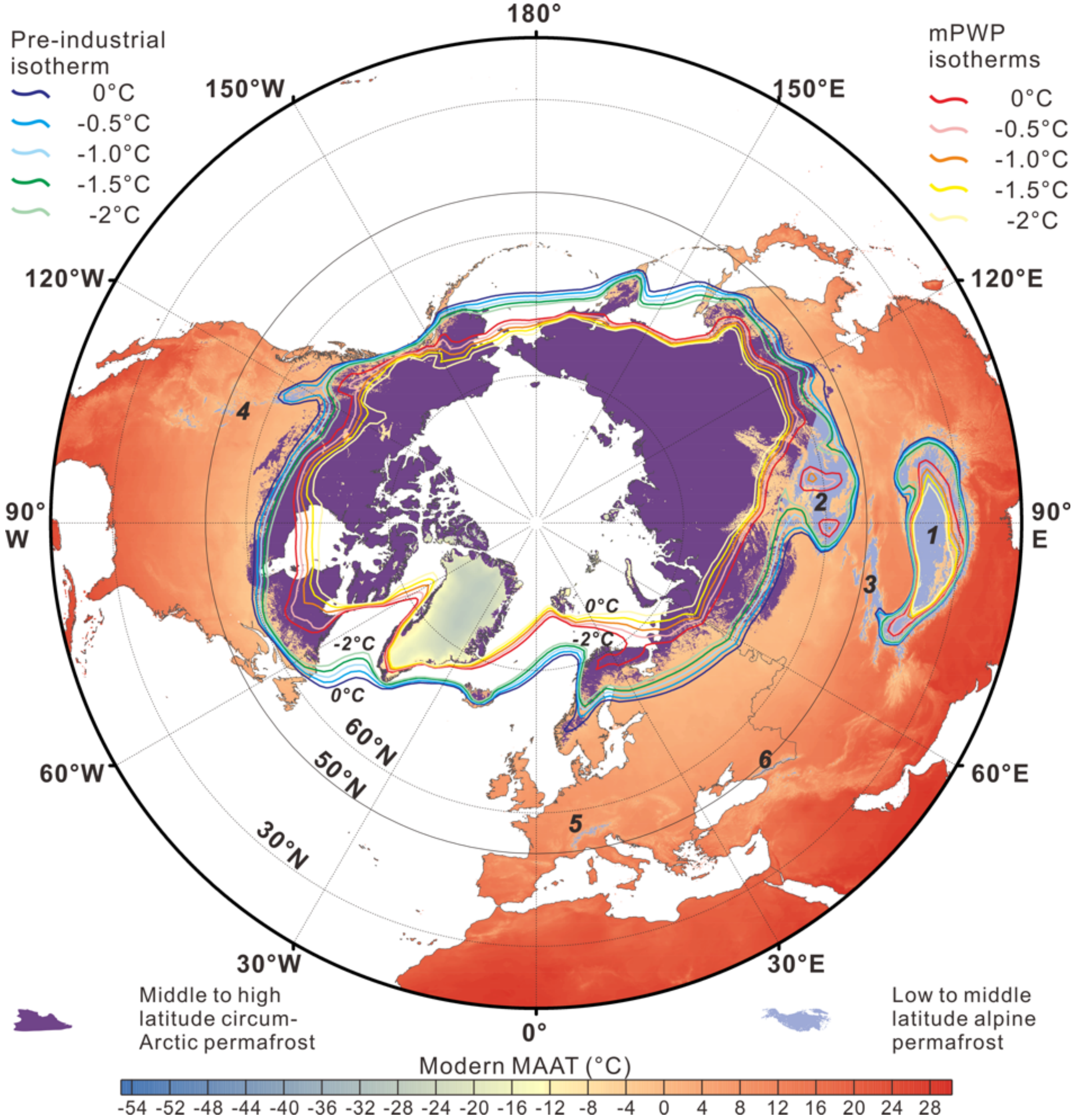


**Supplementary Figure 7. Modern mean annual air temperature (MAAT) and simulated MAAT during the mid-Pliocene Warm Period (mPWP)**. Base map shows the modern MAAT[54]. The mPWP and pre-Industrial isotherms (i.e., -2°C, -1.5°C, -1°C, -0.5°C, and 0°C) are derived from the Pliocene Model Intercomparison Project Phase 2 (PlioMIP2)-based climate model simulation[68]. Note that the pre-industrial 0°C isotherm more accurately reflects the modern permafrost distribution compared with other pre-industrial isotherms. Region marked with 1-6 refers to the Tibetan Plateau-Pamir, Altai Mountains-Mongolia-Yablonoi-Sayan, Tian Shan, Rocky Mountains, Alps, and Caucasus respectively.

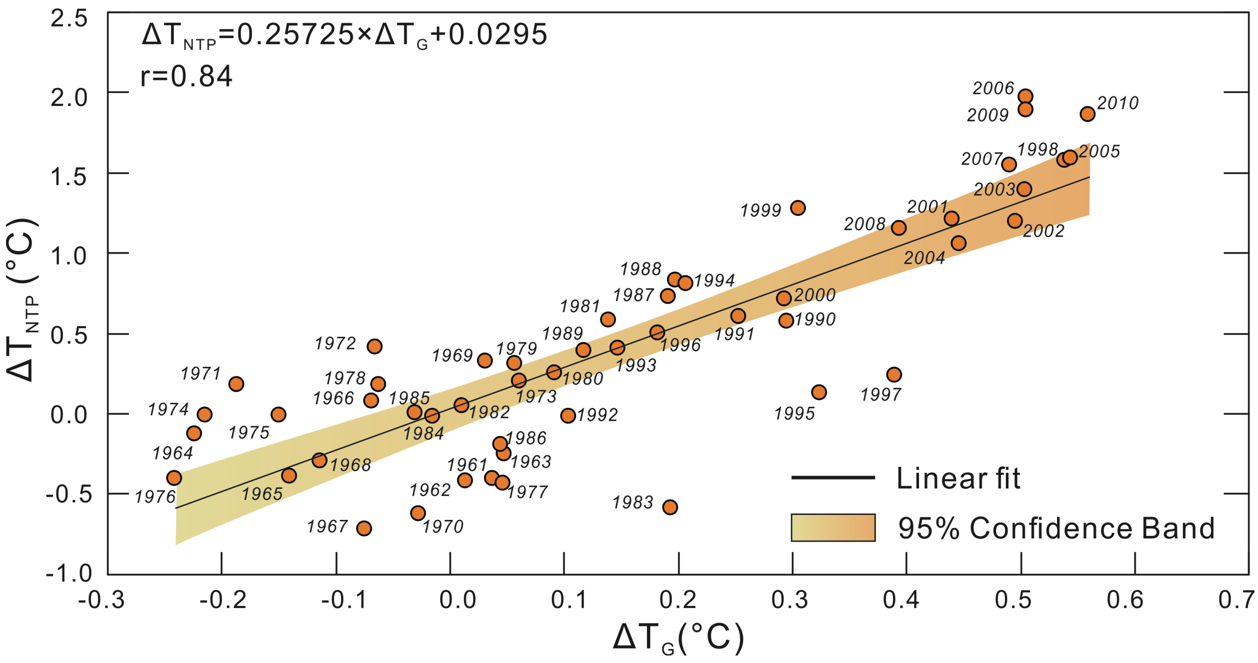


**Supplementary Figure 8. Cross plot of global temperature anomaly** ($\Delta T_G$)[51] **(relative to the of 1961-1990 average) and MAAT anomaly[50] (relative to the 1961-1990 average) in the northern Tibetan Plateau** ($\Delta T_{NTP}$) **for the 1961-2010 period.** Note the linear correlation between the $\Delta T_G$ and $\Delta T_{NTP}$.

**Supplementary Table 1.** Result of student's T-test, showing regional climate change at 2.7 Ma at Kunlun Pass site

| T-test | Age divide (Ma) | $\delta^{13}C$ (VPDB,‰) | $\delta^{18}O$ (VPDB,‰) | $CaCO_3$ (%) | C/N | TOC (%) | TN (%) | $\delta^{13}C_{org}$ (‰) | D (μm) | Surface lake summer temperature (SLST) (°C) | Mean Annual Air Temperature (MAAT)(°C) | Water $\delta^{18}O$ (VSMOW, ‰) |
|---|---|---|---|---|---|---|---|---|---|---|---|---|
| P value | 2.7 | $9.6\times10^{-7}$ | $2.8\times10^{-22}$ | $1.8\times10^{-16}$ | 0.075 | $7.5\times10^{-7}$ | $1.8\times10^{-16}$ | $5.9\times10^{-7}$ | $7.7\times10^{-8}$ | $2.1\times10^{-3}$ | $2.1\times10^{-3}$ | $1.6\times10^{-6}$ |
| | 1.0 | 0.275 | 0.061 | $3.3\times10^{-3}$ | 0.013 | 0.407 | 0.010 | 0.192 | 0.285 | 0.759 | 0.766 | 0.147 |
| | 1.5 | 0.299 | 0.506 | $1.2\times10^{-3}$ | 0.773 | 0.025 | $2.4\times10^{-4}$ | $5.1\times10^{-3}$ | 0.153 | 0.534 | 0.534 | 0.055 |
| | 2.0 | 0.136 | $1.7\times10^{-6}$ | $7.6\times10^{-7}$ | 0.506 | 0.070 | $4.6\times10^{-5}$ | $4.5\times10^{-5}$ | 0.176 | 0.228 | 0.216 | 0.026 |
| | 2.5 | $2.6\times10^{-5}$ | $5.6\times10^{-22}$ | $3.7\times10^{-15}$ | 0.263 | $1.9\times10^{-5}$ | $5.2\times10^{-14}$ | $4.5\times10^{-6}$ | $1.2\times10^{-7}$ | 0.019 | 0.018 | $5.6\times10^{-4}$ |
| | 3.0 | $7.8\times10^{-4}$ | $6.2\times10^{-15}$ | $6.5\times10^{-13}$ | 0.099 | $2.1\times10^{-6}$ | $2.6\times10^{-15}$ | $5.7\times10^{-7}$ | $1.1\times10^{-7}$ | 0.033 | 0.034 | $7.5\times10^{-4}$ |
| | 3.5 | 0.039 | $4.8\times10^{-7}$ | $9.5\times10^{-5}$ | $1.5\times10^{-4}$ | $1.4\times10^{-10}$ | $8.9\times10^{-20}$ | $1.2\times10^{-11}$ | $1.5\times10^{-10}$ | 0.099 | 0.103 | 0.025 |
| | 4.0 | 0.139 | 0.020 | 0.498 | $8.8\times10^{-4}$ | $1.5\times10^{-8}$ | $1.1\times10^{-16}$ | $2.7\times10^{-4}$ | $1.8\times10^{-9}$ | 0.133 | 0.143 | 0.098 |
