# Supplementary material for: Alpine permafrost could account for a quarter of thawed carbon based on Plio-Pleistocene paleoclimate analogue
Source: Nat Commun. 2022 Mar 14;13:1329. doi: 10.1038/s41467-022-29011-2 (PMC8921200; doi:10.1038/s41467-022-29011-2)
Supplement: Supplementary file 2 — Description of Additional Supplementary Files [file 41467_2022_29011_MOESM2_ESM.pdf]

Description of Additional Supplementary Files

File Name: Supplementary Data 1
Description: Results of carbonate ($CaCO_3$) content and carbonate stable isotopes including oxygen ($\delta^{18}O_c$), carbon ($\delta^{13}C_c$), and clumped isotopes (Δ_{47}), as well as grain size and a suite of organic proxies including total nitrogen (TN), total organic carbon (TOC), carbon to nitrogen ratios (C/N), and carbon isotope ratios in organic matter ($\delta^{13}C_{org}$). Clumped isotopes raw data, cross plots of $\delta^{18}O_c$ and Δ_{47}-based temperature, calculations related to mean annual air temperature calculation (MAAT), and surface uplift estimate are also included.

File Name: Supplementary Data 2
Description: Estimate of mean annual air temperature (MAAT) using a different temperature-Δ_{47} calibration for comparison.

File Name: Supplementary Data 3
Description: Calculation of permafrost thawing area and carbon release.
